# A comprehensive reference genome assembly dataset of birds inhabiting Denmark, Greenland, and the Faroe Islands

**DOI:** 10.1093/gigascience/giag078

**Published:** 2026-07-07

**Authors:** Daniel Bilyeli Øksnebjerg, Sascha Dreyer Nielsen, Guangji Chen, Michael J Andersen, Vanessa Almagro, Roberto Ambrosini, Shigeki Asai, Sharon M Birks, Robb T Brumfield, Vojtěch Brlík, Annaïs Carbajal, Theresa M Burg, Sandra Bouwhuis, Shuang Wang, Wanjun Chen, Santiago Claramunt, Fuqiang Lin, Jiaqi Xu, Wei Dai, Scott V Edwards, Mary Margaret Ferraro, Joerns Fickel, Jon Fjeldså, Giulio Formenti, Jérôme Fuchs, Guido Roberto Gallo, Luca Gianfranceschi, Gary R Graves, Grzegorz Grzywaczewski, Wei Han, Tri Haryoko, Ando Haruko, Olof Hellgren, Juan Carlos Illera, Erich D Jarvis, Lars E Johannessen, Arild Johnsen, Ulf S Johansson, Knud Andreas Jønsson, Wei Jiang, Victoria Jayne, Takaharu Kawashima, Adam D Leaché, Fumin Lei, Jan T Lifjeld, Miriam Liedvogel, Yang Liu, Irby J Lovette, Sarah S T Mak, Isao Nishiumi, Michelle F O’Brien, Manabu Onuma, Bent Petersen, Martin Päckert, Marco Pavia, Alivia Price, Javier Quesada, Carsten Rahbek, Andrew Hart Reeve, Alejandro Rico-Guevara, Markus Risch, Vanya G Rohwer, Takema Saitoh, C Jonathan Schmitt, Anna Schnelle, Manuel Schweizer, Simona Secomandi, Paula Serres-Corral, Sara Shopland, Gang Song, Tamás Székely, Ivan J Starikov, Josefin Stiller, Xulan Shen, Zhongyi Sun, Dieter Thomas Tietze, Pavel S Tomkovich, Alfréd Trnka, Araxi O Urrutia, Sölvi Rúnar Vignisson, Kees Wanders, Michael Wink, Kevin Winker, Christopher C Witt, Jack J Withrow, Jessie L Williamson, Glenn Yannic, Nobuyuki Yamaguchi, Zhengwang Zhang, M Thomas P Gilbert, Guojie Zhang, Shaohong Feng, Peter A Hosner

**Affiliations:** Section for Hologenomics, Globe Institute, University of Copenhagen, Øster Farimagsgade 5, Copenhagen 1353, Denmark; Section for Hologenomics, Globe Institute, University of Copenhagen, Øster Farimagsgade 5, Copenhagen 1353, Denmark; Center for Evolutionary and Organismal Biology, Liangzhu Laboratory, Zhejiang University School of Medicine, 866 Yuhangtang Rd, Xihu District, Hangzhou 310058, China; Department of Biology and Museum of Southwestern Biology, University of New Mexico, 302 Washington St SE, Albuquerque, NM 87106, USA; Parc Zoològic de Barcelona, Parc de la Ciutadella, Barcelona 08003, Spain; Department of Environmental Science and Policy, University of Milan, Via Giovanni Celoria 26, Milan 20133, Italy; Biodiversity Genomics Research Centre, University of Milan, Via Giovanni Celoria 26, Milan 20133, Italy; Yamashina Institute for Ornithology,115 Konoyama, Abiko, Chiba 270-1145, Japan; Burke Museum, University of Washington, 4300 15th Ave NE, Seattle WA 98105, USA; Louisiana State University Museum of Natural Science, 119 Foster Hall, Baton Rouge LA 70803, USA; Department of Ecology, Charles University, Viničná 7, Prague 128 44, Czech Republic; Department of Animal Health and Anatomy, Veterinary Faculty, Universitat Autònoma de Barcelona, Campus de la UAB, Barcelona 08193, Spain; University of Lethbridge, 4401 University Drive W, Lethbridge AB T1K 3M4, Canada; Institute of Avian Research “Vogelwarte Helgoland,” An der Vogelwarte 21, Wilhelmshaven 26386, Germany; Center for Evolutionary and Organismal Biology, Liangzhu Laboratory, Zhejiang University School of Medicine, 866 Yuhangtang Rd, Xihu District, Hangzhou 310058, China; BGI Research, Building 11, Beidou Industrial Park, East Lake High-Tech Development Zone, Wuhan, Hubei 430074, China; Department of Biological Sciences, Louisiana State University New Orleans, 2000 Lakeshore Dr, New Orleans LA 70148, USA; Department of Natural History, Royal Ontario Museum, 100 Queens Park, Toronto M5S 2C6, Canada; Center for Evolutionary and Organismal Biology, Liangzhu Laboratory, Zhejiang University School of Medicine, 866 Yuhangtang Rd, Xihu District, Hangzhou 310058, China; Center for Evolutionary and Organismal Biology, Liangzhu Laboratory, Zhejiang University School of Medicine, 866 Yuhangtang Rd, Xihu District, Hangzhou 310058, China; BGI Research, Building 11, Beidou Industrial Park, East Lake High-Tech Development Zone, Wuhan, Hubei 430074, China; Department of Organismic and Evolutionary Biology, Harvard University, 26 Oxford St, Cambridge MA 02138, USA; Museum of Comparative Zoology, Harvard University, 26 Oxford St, Cambridge MA 02138, USA; Cornell University Museum of Vertebrates, Cornell University, 159 Sapsucker Woods Rd, Ithaca NY 14850, USA; Leibniz Institute for Zoo and Wildlife Research, Alfred-Kowalke-Straße 17, Berlin 10315, Germany; Institute for Biochemistry & Biology, University of Potsdam, Karl-Liebknecht-Str. 24-25, Potsdam-Golm D-14476, Germany; Natural History Museum of Denmark, University of Copenhagen, Øster Voldgade 5-7, Copenhagen K 1350, Denmark; The Vertebrate Genome Laboratory, The Rockefeller University, 1230 York Ave, New York NY 10065, USA; Institut de Systématique Evolution Biodiversité, Muséum national d’Histoire Naturelle, CNRS, SU, EPHE-PSL, UA, 57 Rue Cuvier, Paris 75005, France; Biodiversity Genomics Research Centre, University of Milan, Via Giovanni Celoria 26, Milan 20133, Italy; Department of Biosciences, University of Milan, Via Giovanni Celoria 26, Milan 20133, Italy; Biodiversity Genomics Research Centre, University of Milan, Via Giovanni Celoria 26, Milan 20133, Italy; Department of Biosciences, University of Milan, Via Giovanni Celoria 26, Milan 20133, Italy; Department of Vertebrate Zoology, National Museum of Natural History, Smithsonian Institution, 10th St. & Constitution Ave. NW, Washington, DC 20560, USA; Center for Macroecology, Evolution and Climate, Globe Institute, University of Copenhagen, Universitetsparken 15, Copenhagen 2100, Denmark; Department of Zoology and Animal Ecology, University of Life Sciences Lublin, Akademicka 13, 20-950, Lublin, Poland; Center for Evolutionary and Organismal Biology, Liangzhu Laboratory, Zhejiang University School of Medicine, 866 Yuhangtang Rd, Xihu District, Hangzhou 310058, China; Museum Zoologicum Bogoriense, Research Center for Biosystematics and Evolution, National Research and Innovation Agency (BRIN) Jl. Raya Jakarta-Bogor Km. 46, Cibinong, Bogor, West Java 16911, Indonesia; National Institute for Environmental Studies (NIES),16-2 Onogawa, Tsukuba 305-8506, Japan; Evolutionary Ecology and Infection Biology, Department of Biology, Lund University, Sölvegatan 37, Lund 223 62, Sweden; Biodiversity Research Institute (CSIC-Oviedo University-Principality of Asturias), University of Oviedo, Campus of Mieres, Edificio de Investigación, c/ Gonzalo Gutiérrez Quirós s/n, Mieres, Asturias 33600, Spain; The Vertebrate Genome Laboratory, The Rockefeller University, 1230 York Ave, New York NY 10065, USA; Natural History Museum, University of Oslo, Sars' gate 1, Oslo 0562, Norway; Natural History Museum, University of Oslo, Sars' gate 1, Oslo 0562, Norway; Swedish Museum of Natural History, Frescativägen 40, Stockholm 114 18, Sweden; Natural History Museum of Denmark, University of Copenhagen, Øster Voldgade 5-7, Copenhagen K 1350, Denmark; Swedish Museum of Natural History, Frescativägen 40, Stockholm 114 18, Sweden; BGI Research, Building 11, Beidou Industrial Park, East Lake High-Tech Development Zone, Wuhan, Hubei 430074, China; School of Life Sciences, The Chinese University of Hong Kong, Science Centre, CUHK, Shatin, New Territories, Hong Kong SAR, China; National Institute for Environmental Studies (NIES),16-2 Onogawa, Tsukuba 305-8506, Japan; Burke Museum, University of Washington, 4300 15th Ave NE, Seattle WA 98105, USA; Institute of Zoology, Chinese Academy of Sciences, 1 Beichen West Road, Beijing 100101, China; Natural History Museum, University of Oslo, Sars' gate 1, Oslo 0562, Norway; Institute of Avian Research “Vogelwarte Helgoland,” An der Vogelwarte 21, Wilhelmshaven 26386, Germany; State Key Laboratory of Biocontrol, School of Ecology, Sun Yat-sen University, No. 66, Gongchang Road, Shenzhen 518107, China; Cornell University Museum of Vertebrates, Cornell University, 159 Sapsucker Woods Rd, Ithaca NY 14850, USA; Cornell Lab of Ornithology, Cornell University, 159 Sapsucker Woods Rd, Ithaca NY 14850, USA; Section for Hologenomics, Globe Institute, University of Copenhagen, Øster Farimagsgade 5, Copenhagen 1353, Denmark; Department of Zoology, National Museum of Nature and Science, 7-20 Uenokoen, Tokyo 110-8718, Japan; Wildfowl and Wetlands Trust Slimbridge, Slimbridge, Gloucestershire GL2 7BT, UK; National Institute for Environmental Studies (NIES),16-2 Onogawa, Tsukuba 305-8506, Japan; Section for Hologenomics, Globe Institute, University of Copenhagen, Øster Farimagsgade 5, Copenhagen 1353, Denmark; Center of Excellence for Omics-Driven Computational Biodiscovery (COMBio), AIMST University, Jalan Bedong - Semeling, Kedah 08100, Malaysia; Senckenberg Natural History Collections Dresden, Königsbrücker Landstraße 159, Dresden 01109, Germany; Museo di Geologia e Paleontologia, Dipartimento di Scienze della Terra, University of Torino, Via Valperga Caluso 35, Torino 10125, Italy; Department of Biology, University of Copenhagen, Copenhagen, Denmark; Department of Vertebrates, Natural Science Museum of Barcelona, Barcelona, Spain; Center for Macroecology, Evolution and Climate, Globe Institute, University of Copenhagen, Universitetsparken 15, Copenhagen 2100, Denmark; Center for Global Mountain Biodiversity, Globe Institute, University of Copenhagen, Universitetsparken 15, Copenhagen Ø 2100, Denmark; Department of Biology, University of Southern Denmark, Campusvej 55, Odense M 5230, Denmark; Natural History Museum of Denmark, University of Copenhagen, Øster Voldgade 5-7, Copenhagen K 1350, Denmark; Swedish Museum of Natural History, Frescativägen 40, Stockholm 114 18, Sweden; Burke Museum, University of Washington, 4300 15th Ave NE, Seattle WA 98105, USA; Bündnis Naturschutz in Dithmarschen e.V., Meldorfer Straße 17, Hemmingstedt 25770, Germany; Cornell University Museum of Vertebrates, Cornell University, 159 Sapsucker Woods Rd, Ithaca NY 14850, USA; Yamashina Institute for Ornithology,115 Konoyama, Abiko, Chiba 270-1145, Japan; Department of Organismic and Evolutionary Biology, Harvard University, 26 Oxford St, Cambridge MA 02138, USA; Museum of Comparative Zoology, Harvard University, 26 Oxford St, Cambridge MA 02138, USA; Institute of Avian Research “Vogelwarte Helgoland,” An der Vogelwarte 21, Wilhelmshaven 26386, Germany; Natural History Museum Bern, Bernastrasse 15, Bern 3005, Switzerland; Institute of Ecology and Evolution, University of Bern, Baltzerstrasse 6, Bern 3012, Switzerland; Department of Biosciences, University of Milan, Via Giovanni Celoria 26, Milan 20133, Italy; Laboratory of Neurogenetics of Language, The Rockefeller University, 1230 York Ave, New York, NY 10065, USA; Department of Animal Health and Anatomy, Veterinary Faculty, Universitat Autònoma de Barcelona, Campus de la UAB, Barcelona 08193, Spain; Wildfowl and Wetlands Trust Slimbridge, Slimbridge, Gloucestershire GL2 7BT, UK; Institute of Zoology, Chinese Academy of Sciences, 1 Beichen West Road, Beijing 100101, China; Department of Life Sciences, Milner Centre for Evolution, University of Bath, Claverton Down, Bath BA2 7AY, UK; Zoological Institute of Russian Academy of Sciences, Universitetskaya Emb., 1, St., St. Petersburg 199034, Russia; Department of Biology, University of Copenhagen, Copenhagen, Denmark; Center for Evolutionary and Organismal Biology, Liangzhu Laboratory, Zhejiang University School of Medicine, 866 Yuhangtang Rd, Xihu District, Hangzhou 310058, China; Center for Evolutionary and Organismal Biology, Liangzhu Laboratory, Zhejiang University School of Medicine, 866 Yuhangtang Rd, Xihu District, Hangzhou 310058, China; Center of Natural History, Universität Hamburg, Martin-Luther-King-Platz 3, Hamburg 20146, Germany; Zoological Museum of Lomonosov Moscow State University, Bol'shaya Nikitskaya Ulitsa, 2, Moscow 125009, Russia; Department of Biology, University of Trnava, Priemyselná 4, Trnava 918 43, Slovakia; Instituto de Ecología, Universidad Nacional Autónoma de México, Circuito Exterior S/N, Ciudad Universitaria, Coyoacán, CDMX 04510, México; Sudurnes Science and Learning Center, Sandgerði, Iceland; Natural History Museum of Denmark, University of Copenhagen, Øster Voldgade 5-7, Copenhagen K 1350, Denmark; Center for Macroecology, Evolution and Climate, Globe Institute, University of Copenhagen, Universitetsparken 15, Copenhagen 2100, Denmark; Department of Life Sciences, Milner Centre for Evolution, University of Bath, Claverton Down, Bath BA2 7AY, UK; Institute of Pharmacy and Molecular Biotechnology, Heidelberg University, Im Neuenheimer Feld 364, Heidelberg 69120, Germany; University of Alaska Museum, 1962 Yukon Dr, Fairbanks AK 99775, USA; Department of Biology and Museum of Southwestern Biology, University of New Mexico, 302 Washington St SE, Albuquerque, NM 87106, USA; University of Alaska Museum, 1962 Yukon Dr, Fairbanks AK 99775, USA; Cornell University Museum of Vertebrates, Cornell University, 159 Sapsucker Woods Rd, Ithaca NY 14850, USA; Cornell Lab of Ornithology, Cornell University, 159 Sapsucker Woods Rd, Ithaca NY 14850, USA; Department of Zoology and Physiology, University of Wyoming, 1000 E University Ave, Laramie, WY 82071, USA; Université Grenoble Alpes, Université Savoie Mont Blanc, CNRS, LECA, 2233 Rue de la Piscine, Gières 38610, France; Groupe de Recherche en Ecologie Arctique (GREA), 16 rue de Vernot, Francheville 21440, France; Institute of Tropical Biodiversity and Sustainable Development, University Malaysia Terengganu, Malaysia; College of Life Sciences, Beijing Normal University, 19 Xinjiekou Outer St, Beijing 100875, China; Section for Hologenomics, Globe Institute, University of Copenhagen, Øster Farimagsgade 5, Copenhagen 1353, Denmark; Center for Evolutionary and Organismal Biology, Liangzhu Laboratory, Zhejiang University School of Medicine, 866 Yuhangtang Rd, Xihu District, Hangzhou 310058, China; Center for Evolutionary and Organismal Biology, Liangzhu Laboratory, Zhejiang University School of Medicine, 866 Yuhangtang Rd, Xihu District, Hangzhou 310058, China; Department of General Surgery of Sir Run Run Shaw Hospital, Zhejiang University School of Medicine, 3 East Qingchun Road, Hangzhou, Zhejiang 310016, China; Natural History Museum of Denmark, University of Copenhagen, Øster Voldgade 5-7, Copenhagen K 1350, Denmark; Center for Macroecology, Evolution and Climate, Globe Institute, University of Copenhagen, Universitetsparken 15, Copenhagen 2100, Denmark; Center for Global Mountain Biodiversity, Globe Institute, University of Copenhagen, Universitetsparken 15, Copenhagen Ø 2100, Denmark

**Keywords:** Arctic biodiversity, avian genomics, comparative genomics, conservation genetics, evolutionary biology, ecological genomics, wildlife conservation

## Abstract

Reference genome assemblies are essential infrastructure for investigating phylogeny and population/conservation genetics of wild organisms. Birds serve as model vertebrates in ecology and evolutionary biology due to their well-documented natural histories and extensive community science data. We release a set of 350 newly assembled avian genomes, which, when combined with 97 previously published genomes, represent 447 of the bird species recorded in Denmark, the Faroe Islands, and Greenland—the largest regional dataset of a vertebrate group to date. These genomes are published for various research activities. This data release advances the global effort to build comprehensive and accessible biodiversity genomic resources for the research community.

## Introduction

Sequencing and public accessibility of eukaryotic genomes is emerging as a basic need for contemporary and collaborative biological research [1, 2]. These primary datasets provide a foundation for addressing fundamental research questions related to the ecology and evolutionary history of life’s diversity, and the genetic basis of its morphological traits, health, behavior, and other phenotypes. Genomes also provide essential tools for addressing pressing global challenges, including wildlife conservation and adaptation to global change [3].

In light of this need, the B10K avian genome sequencing consortium was founded in 2010 to organize, generate, and release a standardized set of de novo sequenced draft avian genomes of selected species [4]. Previous data releases from the B10K comprise a set of 48 avian genomes in 2014, representing most avian orders [5–7], followed by an additional 267 new genomes in 2020, representing 218 avian families [8]. To complement these data, here we report the release of an additional 350 avian genomes, which, when combined with 97 relevant previously published avian genomes, totals 447 species that largely represent the natural avian species diversity recorded in Denmark, the Faroe Islands, and Greenland. Our rationale for producing this dataset was based on the need to generate comprehensive reference datasets that can be used by national authorities and researchers to assist in conservation-related research. As such, to the best of our knowledge, this data release constitutes the first comprehensive genome dataset of a major vertebrate taxonomic group for any group of nations. These bird species are generally wide-ranging; hence, the data will be broadly relevant for researchers with genomic interests working in Scandinavia, Europe, the Western Palaearctic, and Arctic regions as a whole.

## Methods

### Tissue sample collection and preparation

Tissue samples representing the target species were sourced from museums, research labs, and other biological collections and their associated researchers (Supplementary Table S1). Species names follow The Howard and Moore Complete Checklist of the Birds of the World, version 4.0, the taxonomy authority used by the B10K consortium. We aimed to be as comprehensive in our catalog as possible, including species that have been extirpated from parts of their former distributions or that occur occasionally or rarely as migrants or vagrants. Hence, not all the samples processed were originally collected in Denmark, Greenland, and the Faroe Islands, although we prioritized the sampling of local subspecies and populations when possible.

### Spatial distribution of focal species

To map the utility of the genome set across the Western Palearctic region, we used observation records of the 447 focal bird species obtained from the Global Biodiversity Information Facility (GBIF) to model their geographical distributions. Species distribution models were constructed with the R package “ENMeval” [9] for translating the observational records into spatial ranges. To avoid model overfitting, we only retained 7 of the 19 bioclimatic variables used to construct the models [10]. Each of the 447 individual range models was then overlaid to create a composite map (Supplementary Fig. S1).

### DNA extraction

Genomic DNA was extracted from blood and tissue samples using the “MagMAX DNA Multi-Sample Ultra 2.0” kit (Thermo Fisher) following the manufacturer’s guidelines. Post-lysis, we included an RNase A step at 37°C, followed by bead-based purification and automated isolation on the Kingfisher Duo Prime using the MMX_Ultra2_Cell_Tissue_96_Duo program. Quality control was performed on the 2200 Tapestation System, and all DNA extractions were stored at −80°C until library construction.

### Genome sequencing

#### Sequencing technologies employed

Multiple library construction and sequencing strategies were used to produce raw reads for newly collected samples. The differences in library preparation methods and sequencing platforms used reflect techniques available at the time samples were processed. For 336 samples, MGI’s single-tube Long Fragment Read (st-LFR) technology [11] was used, after which the resulting DNA Nanoballs (DNBTMs) were sequenced with 100-bp paired-end reads (PE100 + 100 + multiple barcode length modes). For eight samples, the 10X Genomics linked-reads technology was used, followed by sequencing on BGIseq-500 with 150-bp paired-end reads (PE150 + 100 + 42 barcode read length). For six samples, standard Next-Generation technology was used to construct small insert size libraries (300–500 bp), which then underwent DNA Nanoball (DNBTM) generation and DNBSeq sequencing with 150-bp paired-end reads (PE150 + 150 + 10 barcode read length). In total, we produced ∼60 Tb of sequencing data for 350 newly released Danish bird species. With an average genome size of 1.2 Gb, the approximate average genome coverage in our dataset is 143 x.

### Bioinformatics analysis

#### Assembly of genomes

For the 336 samples sequenced using st-LFR technology, data were initially transformed into 10X Genomics linked-reads format. Subsequently, this sample set, together with eight samples sequenced by 10X Genomics linked-reads technology, was introduced into the Supernova software (v2.0.1) [13] to assemble the genomes under the “pseudohap” mode. After removing scaffolds with “N” > 80%, gap filling was also done with Gapcloser (v1.12) [12] and the paired-end information as above.

For the six samples sequenced by the standard Next-Generation technology, clean reads were assembled using the SOAPdenovo (v2.04) [12] with a K-mer size of 23-mer following the B10K family-phased assembly strategy [8]. After removing scaffolds with “N” > 80%, GapCloser (v1.12) [12] was used to close the intra-scaffold gaps based on the paired-end information.

#### Gene structures annotation

Annotation of protein-coding genes was conducted with a homology-based method for both the 350 newly sequenced and 97 published Danish bird species following the pipeline implemented by the B10K consortium [8]. The reference gene set consists of the primary reference gene set (20,194 genes), the supplemental human gene set (20,169 genes), and the supplemental transcriptome gene set (5,257 transcripts). The protein sequences in the primary reference gene set were first aligned to each genome using TBLASTN (v2.2.26) [15] with an e-value cut-off 1e-5, and multiple adjacent hits of the same query were connected by genBlastA (v1.0.4) [16]. Homologous blocks with a length greater than 30% of the query protein length were retained. Each connected hit region was later extended to include its 2-kb upstream and downstream flanking regions, on which gene structure was predicted by Genewise (v2.4.1) [17]. MUSCLE (v3.8.31) [18] was then used to align the annotated protein against its reference protein. Predicted proteins with length ≥30 amino acids and an identity value ≥40% were retained. Pseudogenes (annotated genes containing >2 frameshifts or >1 premature stop codon) and retrogenes were further removed. Next, gene models that overlapped in >40% of their coding sequence were clustered into one group and the one with the highest identity to the reference proteins was retained to form a non-redundant gene set for each species. Two supplemental gene sets were also used for homology-based gene prediction for these newly released assemblies as above, but only the newly annotated loci from these supplemental sets were kept. Finally, all candidate annotated genes that had >10 duplications, or were single-exon genes yet contained >70% repeat sequences in the coding region, were removed.

#### Repeats annotation

Tandem repeats were identified by Tandem Repeat Finder (v4.09) [19], and transposable elements (TEs) were annotated using a combination of homology-based RepeatMasker (v4.1.2), and de novo methods with RepeatModeler (v2.0.2a) [20] and LTR_Finder (v1.07) [20, 21]. The homology-based annotation of TEs was performed by RepeatMasker with parameters “-nolow -no_is -norna -engine ncbi -parallel 1” at the DNA level with its built-in library. The de novo repeat annotation of all 447 species was done by RepeatModeler and LTR_Finder with default parameters to first build a de novo repeat library for each assembly, which was further used by RepeatMasker to predict repeats for each species. The *Ficedula albicollis* (NCBI:txid59894) genome (GCA_000247815.2) was omitted due to an undeterminable error during the annotation of repeats, and the other genomes were sufficient for comparison.

### Overview of 447 genomes

#### Species diversity

We obtained fresh tissue samples for all 497 species recorded in Denmark and all breeding birds of the Faroe Islands and Greenland, save two extinct species, when we ceased sample collection in 2023 (Supplementary Fig. S1). However, not all extractions were successful in isolating high-molecular-weight DNA. When samples failed to yield adequate DNA quality and quantity, we sought replacement samples. Yet for some species, no replacement was found, or obtaining and sequencing a replacement would have delayed the data release substantially.

The total genome dataset therefore comprises 447 species. This includes the vast majority of the 497 bird species recorded in Denmark up to 2003 (BirdLife Denmark). It also includes most regular breeding species and regular visitors to Greenland and the Faroe Islands. The majority of the missing species (30) are vagrants, rare visitors, and migrants that have only been sporadically recorded in Denmark. Twenty of the missing species are regularly occurring. Additionally, the dataset reflects avian taxonomic diversity well. All bird orders and families occurring in the target regions are represented.

#### Genome statistics

**Figure 1:**
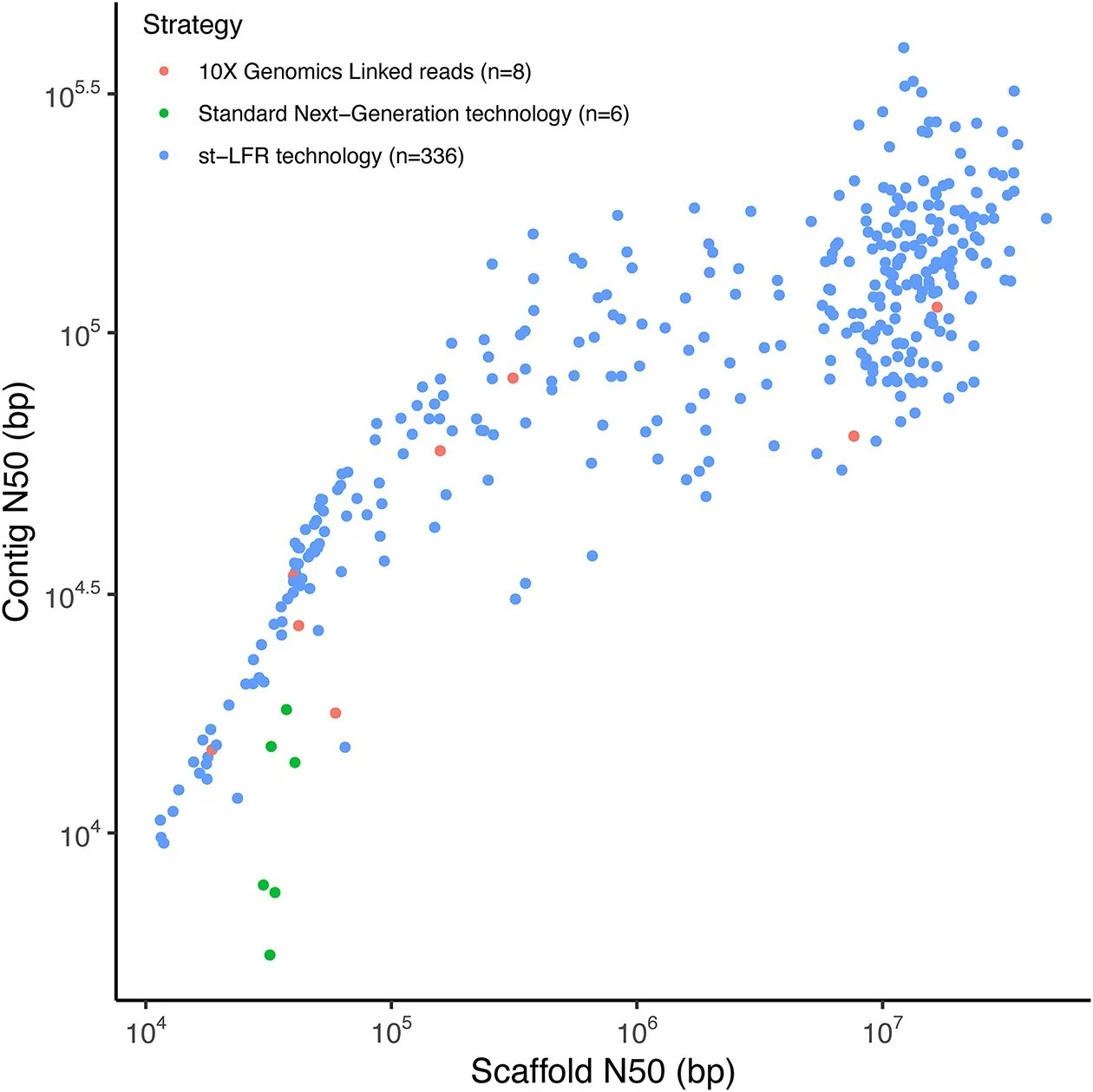
Assembly statistics for 350 newly released avian genomes are colored by each of the three sequencing strategies used.

**Figure 2:**
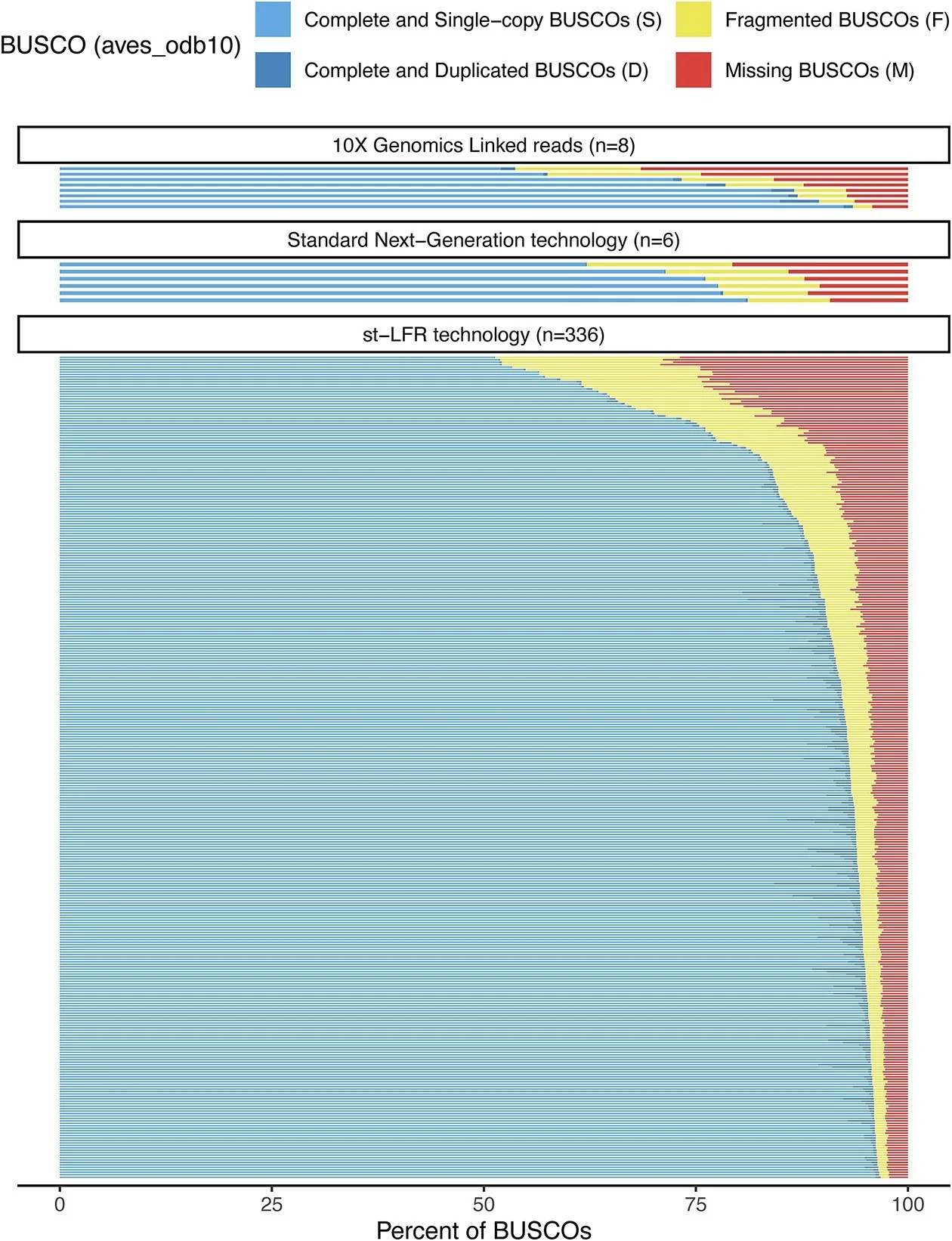
BUSCO evaluation for 350 newly released avian genomes of the birds inhabiting Denmark, Greenland, and the Faroe Islands.

**Figure 3:**
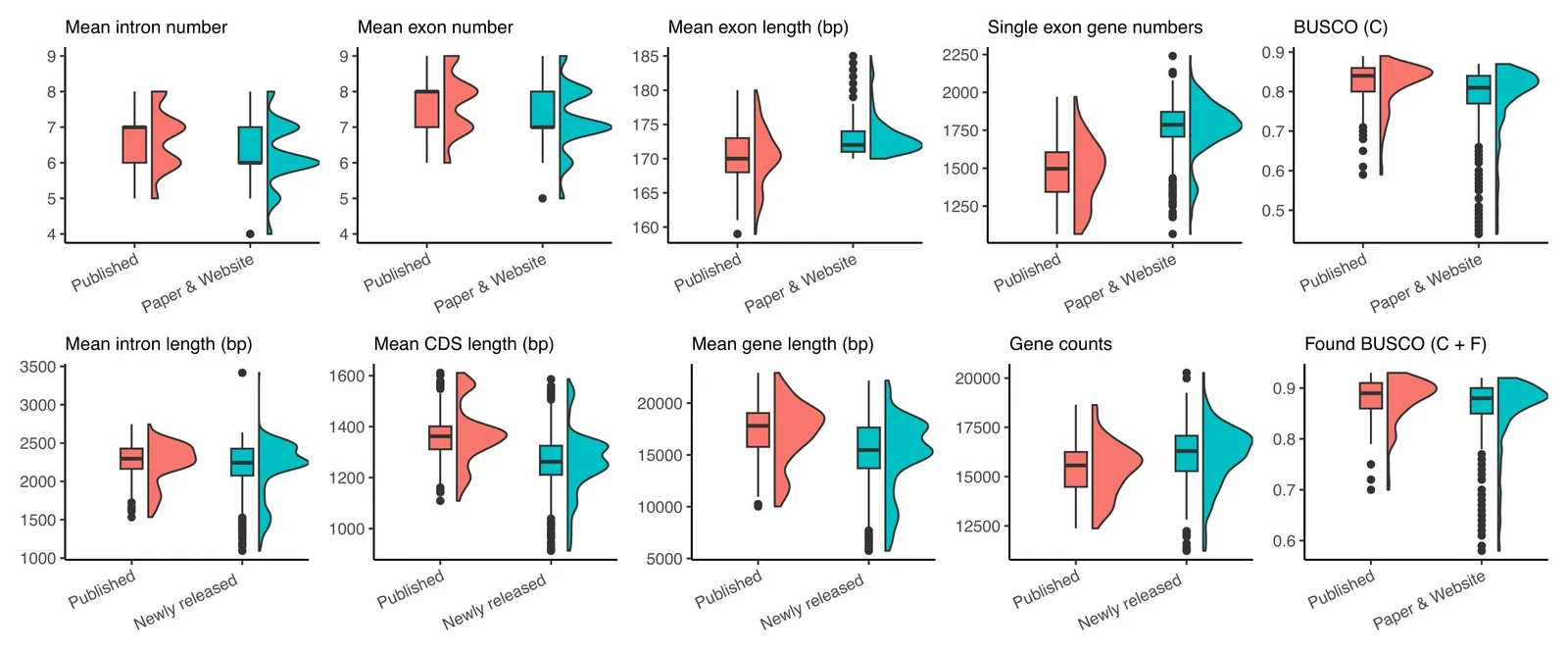
Ten indicators of gene annotation statistics for the 350 newly released avian genomes. The y-axis measures the number of occurrences; box plots, and density distributions show the distribution of values across the genomes.

**Figure 4:**
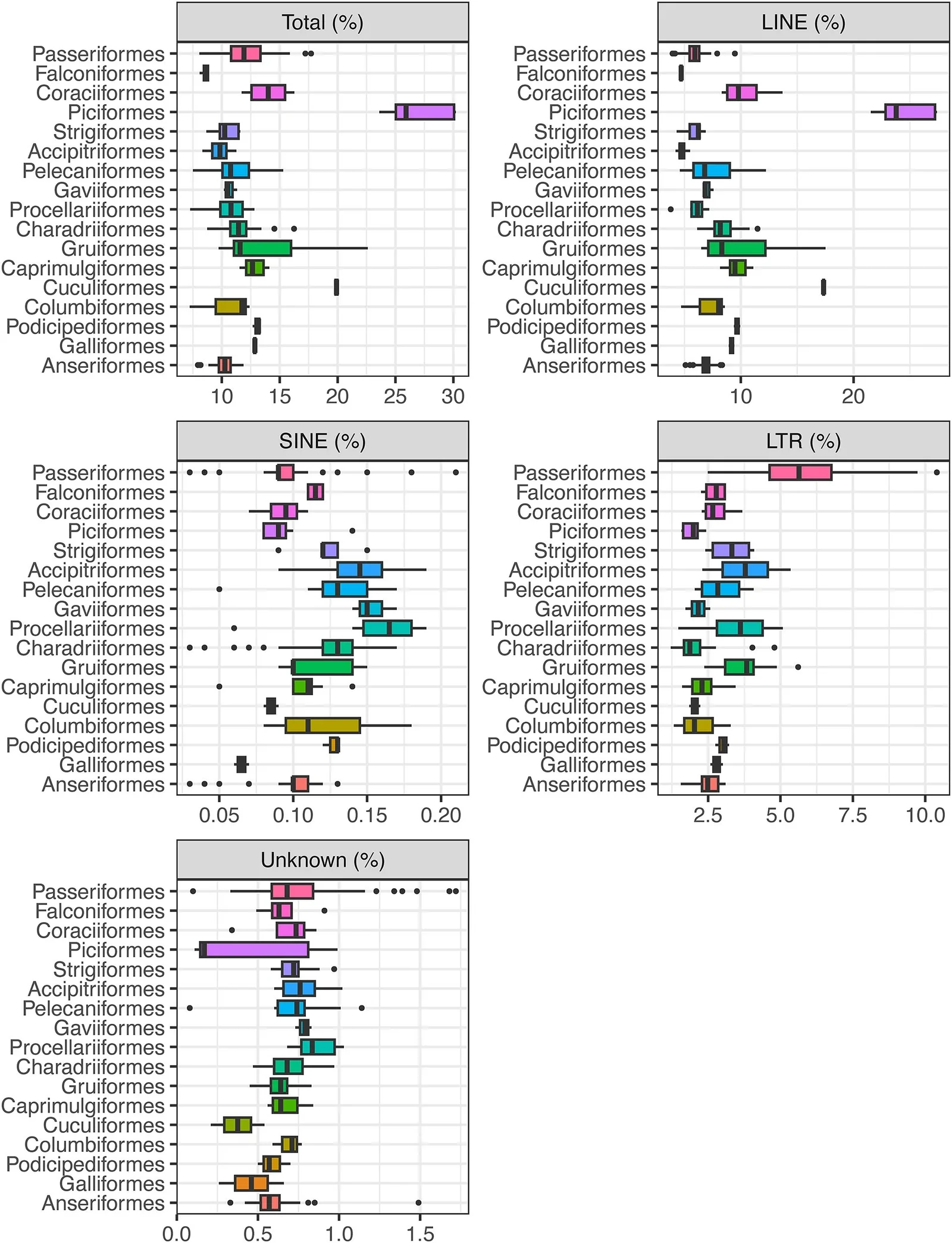
Transposable element (TE) percentages for the 350 newly released avian genomes. Box plots are shown for orders with at least three sequenced species.

The genome assemblies of the 350 newly released avian species ranged from 1.0 to 1.4 Gb, with average scaffold N50 = 8.2 Mb, and contig N50 = 105.3 kb (Fig. 1; Supplementary Figs S2–S4). Only 6.4% of the core genes in aves_odb10 could not be predicted on these newly released genomes (ranging from 2.2% to 31.5%), suggesting that the completeness of these genomes was suitable for most comparative genome analyses (Fig. 2; Supplementary Fig. S5). An average of 89.1% complete core genes in the newly released genomes was comparable to that of the previously published genomes in the dataset (90.4%), but slightly less than bird genomes produced to VGP standards (92–95%, [22]). Duplication rates inferred from the BUSCO measurements varied between 0% and 10.1% among the genomes. On average, only 4.6% of the core genes were partly annotated for the 350 new genomes. Using the homologous annotation method, the 447 genomes were predicted to contain an average of 15,915 protein-coding genes (ranging from *Clangula hyemalis*: 11,235 to *Chroicocephalus ridibundus*: 20,269), similar to previously published bird genomes [8]. The average gene length and coding sequence length are ∼154.6 and 1.3 kb, respectively. Genes contained on average ∼7 exons, with an average length of 173 bp and an average intron length of 2.2 kb (Fig. 3; Supplementary Figs S6–S8). Consistent with the results of previous studies [5, 8], the genomes of these newly sequenced species contained a low proportion of TEs (11.9% on average), except for eight outliers (all species of woodpeckers in Piciformes; Fig. 4; Supplementary Fig. S9). While nearly all analyzed genomes exhibited similar repeat content, they varied in content for each category. For all species, the most abundant repeat category was LINE elements (7.2% on average). Northern wryneck (*Jynx torquilla*) has the richest LINE repeats of 27.4%, while yellow-nosed albatross (*Thalassarche chlororhynchos*) has the richest SINE repeats of 0.23%. Lapland longspur (*Calcarius lapponicus*) had the most genome sequences identified as LTR (10.4%). Of the newly released genomes, 53% are from ZW females and 47% are from ZZ males; the latter lack draft W chromosome scaffolds.

## Data validation and quality control

### Sample verification

Voucher specimens were verified against reference collections. Mitogenomes were BLAST searched against references in the NCBI database. If no reference mitogenome was available, we checked NCBI for commonly sequenced mitochondrial genes (COI, ND2, and CYB) as a reference to verify sequence authenticity. For genomes that lacked a mitochondrial assembly, which was frequent when blood was the source tissue, we used a collection of nuclear markers well-represented on NCBI for birds (FGB, GAPDH, MB, MUSK, ODC, RAG1, and TGFb2).

### Genome sequencing

Quality control steps for raw reads before assembly using the SOAPfilter2 package (v2.2) [12] were:

1. Remove reads with more than 10% of N bases.
2. Remove reads with more than 40% low-quality bases (Phred score ≤ 10).
3. Remove reads with undersized insert size.
4. Filter out the PCR duplicates.

### Genome assembly

Assembly quality of both the 350 newly sequenced and the 97 previously published species was assessed by contig N50, scaffold N50, and total assembly length. Genome completeness was measured with BUSCO (v5.4.6) [14] using aves_odb10 as the reference gene set for these species. Three standard categories of BUSCO results were assessed as follows:

1. Complete and single-copy BUSCOs (S)
2. Complete and duplicated BUSCOs (D)
3. Fragmented BUSCOs (F)

The complete (S and D) and fragmentary (F) hits were combined to assess the degree of genome completeness. Only 24 species had genomes with <80% completeness, the lowest being 68.5%. For the six short insert library species, the BUSCO completeness was lower, and percentages ranged from 79.3% to 90.8%.

### Reuse potential

We created this dataset to support future research on birds, especially those important to the biodiversity of Denmark, Greenland, and the Faroe Islands. While it will be useful for broad avian evolutionary studies, we expect its most lasting value will be in conservation genetics and management of the species included. Potential uses include population genomics, mapping genetic structure and gene flow, identifying hybrid zones, studying local adaptation, and investigating disease ecology.

As sequencing technologies continue to improve (and associated costs decrease), the quality of genomes that can be sequenced and de novo assembled continues to improve. The quality of the genomes released here represents a level that was feasible at the time of this project, given the financial, technical, and biosample constraints available to the project. A future aim is to continue improving quality, e.g., by incorporating additional technologies, such as long reads and scaffolding tools like Hi-C, as implemented in initiatives such as the Vertebrate Genome and Darwin Tree of Life Projects [22]. Nevertheless, we believe the quality will be suitable for many of the above possible end uses.

## Conclusion

A key challenge in biodiversity genomics is obtaining tissue samples with high-quality, high-molecular-weight DNA. For this project, we prepared libraries from samples with DNA fragments longer than 10,000 bp. However, many libraries with fragments of 10,000–20,000 bp failed to produce genome assemblies of acceptable quality, and of those that did, benchmark BUSCO scores suffered. For stLFR sequencing, DNA fragments over 60,000 bp are ideal, yet many of our samples—particularly from salvaged birds found dead (e.g., collision victims)—fell short of this standard. These salvaged specimens had a success rate of ~50%, while freshly collected museum specimens and blood samples had success rates exceeding 90%. While salvaged specimens are valuable for collection building and short-read sequencing, their average poorer DNA quality limits their usefulness for de novo genome assemblies, and they should be considered as a secondary option.

Although our release of avian genomes is comprehensive, it is not complete. When completing the project, we made practical decisions about the final dataset because the genomes of some relatively common species produced substandard assemblies. We flag notable absences in our dataset from the Danish avifauna and highlight alternative genomic resources, including garganey (*Spatula querquedula*; Iridian genomes GCA_032191525.1; NCBI:txid75856), black-necked grebe (*Podiceps nigricollis* in prep; DTOL; NCBI:txid85099), wood pigeon (*Columba palumbus*; DTOL GCA_965250375.1; NCBI:txid8934), black-throated loon (*Gavia arctica*; NCBI:txid57069), European herring gull (*Larus argentatus*; DTOL GCA_964417175.1; NCBI:txid35669), short-eared owl (*Asio flammeus*; NCBI:txid56267), sand martin/bank swallow (*Riparia riparia*; Institute of Ecology and Evolution University of Bern, GCA_020917445.1; NCBI:txid88110), black redstart (*Phoenicurus ochruros*; Kent State University GCA_050234705.1; NCBI:txid358815), and yellowhammer (*Emberiza citrinella*; in prep, DTOL; NCBI:txid37595). In addition, one Faroese breeder, European shag (*Phalacrocorax aristotelis*; DTOL GCA_949628215.1; NCBI:txid126867), and one Greenlandic breeder, snow goose (*Anser caerulescens*; Iridian Genomes GCA_051253075.1; NCBI:txid8849), remain targets for future work. Finally, during manuscript preparation, we identified that our genome of the common moorhen (*Gallinula chloropus*; NCBI:txid9123) pertains to the common gallinule (*G. galeata*; NCBI:txid1109398), following a recent taxonomic change. However, we note that a true *G. chloropus* assembly is available from the Sanger Institute’s Darwin Tree of Life Project GCA_964237585.1. We intend to identify additional high-quality samples to fill these gaps as the B10K consortium continues its aims.

## Availability of source code and requirements

Project name: DanishBirdsGenome

Project home page: https://github.com/ChenGuangji/DanishBirdsGenome

License: MIT license

Operating system(s): Cross platform

Programming language: Python, Perl, R, Unix shell

Genome assembly pipeline: https://github.com/BGI-Qingdao/stlfr2supernova_pipeline

Genome annotation pipeline: https://github.com/B10KGenomes/annotation

Statistics and visualizations: https://github.com/ChenGuangji/DanishBirdsGenome

## Additional files

**Supplementary Fig. S1**. Overlapping spatial distribution of the 447 species represented in the dataset, illustrating broad coverage of the Western Palearctic avifauna.

**Supplementary Fig. S2**. Assembly statistics for 350 newly released avian genomes.

**Supplementary Fig. S3**. Assembly statistics for all 447 avian genomes, published and new (paper and website).

**Supplementary Fig. S4**. Assembly statistics for all 447 avian genomes.

**Supplementary Fig. S5**. BUSCO evaluation for all 447 avian genomes.

**Supplementary Fig. S6**. Eight indicators of gene annotation statistics for 350 newly released avian genomes across orders. Box plots are shown for orders with at least three sequenced species.

**Supplementary Fig. S7**. Eight indicators of gene annotation statistics for all 447 avian genomes.

**Supplementary Fig. S8**. Eight gene annotation statistics for all 447 avian genomes across orders. Box plots are shown for orders with at least three sequenced species.

**Supplementary Fig. S9**. Transposable element (TE) percentage for all 447 avian genomes. Box plots are shown for orders with at least three sequenced species.

## Abbreviations

GBIF: Global Biodiversity Information Facility; st-LFR: single-tube Long Fragment Read; TE: transposable element.

## Guidelines for data usage and citation

This data release paper and the GenBank accession information should be cited in relevant publications. The B10K consortium is actively working on large-scale, higher-level avian phylogenomic projects incorporating data released herein. We request that authors with similar research interests contact the B10K steering committee rather than opting to pursue potentially competing projects.

## Declarations

All field sampling efforts followed appropriate local permission structures, laws, and regulations, including the Nagoya Protocol and Institutional Review Boards where they apply. All tissue samples were received and processed at Globe Institute, University of Copenhagen, which is certified to receive and process biological tissues under a permit for Animal By-Products DK-123-oth-905637 issued by the Danish Veterinary and Food Administration. CITES samples received from outside the European Union are under the CITES Scientific Exchange Exemption, registration number DK 014 issued by the Danish Environmental Protection Agency.

## Supplementary Material

giag078_Supplemental_Files

giag078_Authors_Response_To_Reviewer_Comments_Original_Submission

giag078_GIGA-D-25-00401_original_submission

giag078_GIGA-D-25-00401_revision_1

giag078_Reviewer_1_Report_Original_Submission Reviewer 1 -- 11/28/2025

giag078_Reviewer_2_Report_Original_Submission Reviewer 2 -- 12/17/2025

## Data Availability

Genome sequencing data, genome assemblies, and annotations of the 350 species generated in this study have been deposited in the European Nucleotide Archive (ENA) under accession ERP190789. The supplementary material [23] has been deposited in the GigaScience GigaDB database [24]. Sample information for each genome and genome statistics can also be viewed online at [25]. The study used 97 publicly available assemblies from NCBI with the Genome Accession IDs listed in Supplementary Table S1. The reference genomes, gene sets, and published RNA-sequencing data used in the gene annotation and alignment construction of this study are available from Ensembl and NCBI. The database used in the transposable elements annotation is available online [26].
