# Supplementary material for: A comprehensive reference genome assembly dataset of birds inhabiting Denmark, Greenland, and the Faroe Islands
Source: Gigascience. 2026 Jul 7;15:giag078. doi: 10.1093/gigascience/giag078 (PMC13492214; doi:10.1093/gigascience/giag078)
Supplement: Supplementary file 3 [file giag078_giga-d-25-00401_revision_1.pdf]

GigaScience

A comprehensive reference genome assembly dataset of birds inhabiting Denmark, Greenland, and the Faroe Islands

--Manuscript Draft--

**Manuscript Number:**	GIGA-D-25-00401R1
**Full Title:**	A comprehensive reference genome assembly dataset of birds inhabiting Denmark, Greenland, and the Faroe Islands
**Article Type:**	Data Note
**Funding Information:**	Villum Fonden (25900) — Dr Guojie Zhang<br>Villum Fonden (25925) — Dr Carsten Rahbek<br>Danmarks Grundforskningsfond (DNRF143) — Dr Thomas Gilbert<br>Key Technologies Research and Development Program (2023YFA1800500) — Dr Shaohong Feng<br>Danmarks Frie Forskningsfond (1054-00039B) — Dr Peter Hosner
**Abstract:**	Background: Reference genome assemblies are essential infrastructure for investigating phylogeny, and population/conservation genetics of wild organisms. Birds serve as model vertebrates in ecology and evolutionary biology due to their well-documented natural histories and extensive community science data. Findings: We release a set of 350 newly assembled avian genomes, which, when combined with 97 previously published genomes, represent 447 of the bird species recorded in Denmark, the Faroe Islands, and Greenland— the largest regional dataset of a vertebrate group to date. These genomes are published for various research activities. Conclusions: This data release advances the global effort to build comprehensive and accessible biodiversity genomic resources for the research community.
**Corresponding Author:**	Peter Hosner, Ph.D<br>University of Copenhagen: Kobenhavns Universitet<br>Copenhagen, DENMARK
**Corresponding Author Secondary Information:**	
**Corresponding Author's Institution:**	University of Copenhagen: Kobenhavns Universitet
**Corresponding Author's Secondary Institution:**	
**First Author:**	Daniel Øksnebjerg
**First Author Secondary Information:**	
**Order of Authors:**	Daniel Øksnebjerg
	Sascha Dreyer
	Guangji Chen
	Michael Andersen
	Vanessa Almagro
	Roberto Ambrosini
	Shigeki Asai
	Sharon Birks
	Robb Brumfield
	Vojtěch Brlík

Powered by Editorial Manager® and ProduXion Manager® from Aries Systems Corporation

	Annaïs Carbajal
	Theresa Burg
	Sandra Bouwhuis
	Shuang Wang
	Wanjun Chen
	Santiago Claramunt
	Fuqiang Lin
	Jiaqi Xu
	Scott Edwards
	Mary Margaret Ferraro
	Joerns Fickel
	Jon Fjeldså
	Giulio Formenti
	Jerome Fuchs
	Guido Roberto Gallo
	Luca Gianfranceschi
	Gary Graves
	Grzegorz Grzywaczewski
	Wei Han
	Tri Haryoko
	Ando Haruko
	Olof Hellgren
	Juan Carlos Illera
	Erich Jarvis
	Lars Erik Johannessen
	Arild Johnsen
	Ulf Johansson
	Knud Jønsson
	Wei Jiang
	Victoria Jayne
	Takaharu Kawashima
	Adam Leaché
	Fumin Lei
	Jan Terje Lifjeld
	Miriam Liedvogel
	Yang Liu
	Irby Lovette
	Sarah Mak
	Isao Nishiumi
	Michelle O'Brien
	Manabu Onuma

Powered by Editorial Manager® and ProduXion Manager® from Aries Systems Corporation

	Bent Petersen
	Martin Päckert
	Marco Pavia
	Alivia Price
	Javier Quesada Lara
	Carsten Rahbek
	Andrew Reeve
	Alejandro Rico-Guevera
	Markus Risch
	Vanya Rohwer
	Takema Saitoh
	C. Jonathan Schmitt
	Anna Schnelle
	Manuel Schweizer
	Simona Secomandi
	Paula Serres-Corral
	Sara Shopland
	Gang Song
	Tamas Szekely
	Ivan Starikov
	Josefin Stiller
	Xulan Shen
	Zhongyi Sun
	Dieter Thomas Tietze
	Pavel Tomkovich
	Alfred Trnka
	Araxi Urrutia
	Solvi Vignisson
	Kees Wanders
	Michael Wink
	Kevin Winker
	Christopher Witt
	Jack Withrow
	Jessie Williamson
	Glenn Yannic
	Nobuyuki Yamaguchi
	Zhengwang Zhang
	Thomas Gilbert
	Guojie Zhang
	Shaohong Feng
	Peter Hosner, Ph.D

Powered by Editorial Manager® and ProduXion Manager® from Aries Systems Corporation

**Order of Authors Secondary Information:**	
**Response to Reviewers:**	Responses to all reviewer comments are attached in a .docx document "DGB response to reviewers".
**Additional Information:**	
**Question**	**Response**
Are you submitting this manuscript to a special series or article collection?	No
**Experimental design and statistics**<br><br>Full details of the experimental design and statistical methods used should be given in the Methods section, as detailed in our Minimum Standards Reporting Checklist. Information essential to interpreting the data presented should be made available in the figure legends.<br><br>Have you included all the information requested in your manuscript?	Yes
**Resources**<br><br>A description of all resources used, including antibodies, cell lines, animals and software tools, with enough information to allow them to be uniquely identified, should be included in the Methods section. Authors are strongly encouraged to cite Research Resource Identifiers (RRIDs) for antibodies, model organisms and tools, where possible.<br><br>Have you included the information requested as detailed in our Minimum Standards Reporting Checklist?	Yes
**Availability of data and materials**<br><br>All datasets and code on which the conclusions of the paper rely must be either included in your submission or deposited in publicly available repositories (where available and ethically appropriate), referencing such data using a unique identifier in the references and in	Yes

the “Availability of Data and Materials” section of your manuscript.<br><br>Have you have met the above requirement as detailed in our Minimum Standards Reporting Checklist?	
GigaScience has policies and guidelines in place for the use of generative AI-writing tools such as ChatGPT. If you have used such writing tools to assist with writing the manuscript this must be declared and cited in the text. Authors should not list AI-writing tools and other AI-assisted technologies as an author or co-author and should acknowledge that they are fully responsible for text generated or refined by AI-writing tools.<p><br><br>A summary of use (particularly in the introduction or among methods) needs to be included at the end of the paper, and the outputs should also be included as a supplementary file hosted in GigaDB or other open repositories. Please read our guidelines for more information. <p><br><br>By submitting to GigaScience, you are aware of the journal's AI-writing tools policy, and if you have declared use of such tools below, you have acknowledged this where appropriate in your manuscript and have made a summary of use and outputs available. <p><br><b>AI-assisted writing tools have been used in the preparation of this manuscript?	No

Powered by Editorial Manager® and ProduXion Manager® from Aries Systems Corporation

Manuscript

Click here to access/download;Manuscript;Danish_bird_genome_release_ma

Gigascience (data note) aimed at releasing DBG data

A comprehensive reference genome assembly dataset of birds inhabiting Denmark, Greenland, and the Faroe Islands

Daniel Bilyeli Øksnebjerg[1*], Sascha Dreyer Nielsen[1*], Guangji Chen[2*], Michael J. Andersen[3], Vanessa Almagro[4], Roberto Ambrosini[5,6], Shigeki Asai[7], Sharon M. Birks[8], Robb T. Brumfield[9], Vojtěch Brlík[10], Annaïs Carbajal[11], Theresa M. Burg[12], Sandra Bouwhuis[13], Shuang Wang[2], Wanjun Chen[14], Santiago Claramunt[15,16], Fuqiang Lin[2], Jiaqi Xu[2], Wei Dai[14], Scott V. Edwards[17,18], Mary Margaret Ferraro[19], Joerns Fickel[20], Jon Fjeldså[21], Giulio Formenti[22], Jérôme Fuchs[23], Guido Roberto Gallo[6,24], Luca Gianfranceschi[6,24], Gary R. Graves[25,26], Grzegorz Grzywaczewski[27], Wei Han[2], Tri Haryoko[28], Ando Haruko[29], Olof Hellgren[30], Juan Carlos Illera[31], Erich D. Jarvis[22], Lars E. Johannessen[32], Arild Johnsen[32], Ulf S. Johansson[33], Knud Andreas Jønsson[21,33], Wei Jiang[14], Victoria Jayne[34], Takaharu Kawashima[29], Adam D. Leaché[8], Fumin Lei[35], Jan T. Lifjeld[30], Miriam Liedvogel[13], Yang Liu[36], Irby J. Lovette[19,37], Sarah S.T. Mak[1], Isao Nishiumi[38], Michelle F. O'Brien[39], Manabu Onuma[29], Bent Petersen[1,40], Martin Päckert[41], Marco Pavia[42], Alivia Price[43], Javier Quesada[44], Carsten Rahbek[26,45,46], Andrew Hart Reeve[21,33], Alejandro Rico-Guevara[8], Markus Risch[47], Vanya G. Rohwer[19], Takema Saitoh[7], C. Jonathan Schmitt[17,18], Anna Schnelle[13], Manuel Schweizer[48,49], Simona Secomandi[24,50], Paula Serres-Corral[11], Sara Shopland[39], Gang Song[35], Tamás Székely[51], Ivan J. Starikov[52], Josefin Stiller[43], Xulan Shen[2], Zhongyi Sun[2], Dieter Thomas Tietze[53], Pavel S. Tomkovich[54], Alfréd Trnka[55], Araxi O. Urrutia[56,], Solvi Runar Vignisson[57], Kees Wanders[21,26,51], Michael Wink[58], Kevin Winker[59], Christopher C. Witt[3], Jack J. Withrow[59], Jessie L. Williamson[19,37,60], Glenn Yannic[61,62], Nobuyuki Yamaguchi[63], Zhengwang Zhang[64], M. Thomas P. Gilbert[1*], Guojie Zhang[2*], Shaohong Feng[2,65*#], Peter A. Hosner[21,26,45*#]

Affiliations:

[1] Section for Hologenomics, Globe Institute, University of Copenhagen, Copenhagen Denmark
[2] Center for Evolutionary and Organismal Biology, Liangzhu Laboratory, Zhejiang University School of Medicine, Hangzhou 310058, China
[3] Department of Biology and Museum of Southwestern Biology, University of New Mexico, Albuquerque, USA
[4] Parc Zoològic de Barcelona, Barcelona, Spain
[5] Department of Environmental Science and Policy, University of Milan, Milan, Italy
[6] Biodiversity Genomics Research Centre, University of Milan, Milan, Italy
[7] Yamashina Institute for Ornithology, Abiko, Japan
[8] Burke Museum, University of Washington, Seattle, USA
[9] Louisiana State University Museum of Natural Science, Baton Rouge, USA
[10] Department of Ecology, Charles University, Prague, Czech Republic
[11] Department of Animal Health and Anatomy, Veterinary Faculty, Universitat Autònoma de Barcelona, Barcelona, Spain
[12] University of Lethbridge, Lethbridge, Canada
[13] Institute of Avian Research "Vogelwarte Helgoland", Wilhelmshaven, Germany
[14] BGI Research, Wuhan 430074, China.

[15] Department of Biological Sciences, University of New Orleans, New Orleans, USA
[16] Department of Natural History, Royal Ontario Museum, Toronto, Canada
[17] Department of Organismic and Evolutionary Biology, Harvard University, Cambridge, USA
[18] Museum of Comparative Zoology, Harvard University, Cambridge, USA
[19] Cornell University Museum of Vertebrates, Cornell University, Ithaca, USA
[20] Leibniz Institute for Zoo and Wildlife Research, Berlin, Germany
[21] Natural History Museum of Denmark, University of Copenhagen, Copenhagen, Denmark
[22] The Vertebrate Genome Laboratory, The Rockefeller University, New York, USA
[23] Institut de Systématique Evolution Biodiversité, Muséum national d'Histoire Naturelle, CNRS, SU, EPHE-PSL, UA, 75005 Paris, France
[24] Department of Biosciences, University of Milan, Milan, Italy
[25] Department of Vertebrate Zoology, National Museum of Natural History, Smithsonian Institution, Washington DC, USA
[26] Center for Macroecology, Evolution and Climate, Globe Institute, University of Copenhagen, Copenhagen, Denmark
[27] Department of Zoology and Animal Ecology, University of Life Sciences Lublin, Lublin, Poland
[28] Museum Zoologicum Bogoriense, Research Center for Biosystematics and Evolution, National Research and Innovation Agency (BRIN), Cibinong, Indonesia
[29] National Institute for Environmental Studies (NIES), Tsukuba, Japan
[30] Evolutionary Ecology and Infection Biology, Department of Biology, Lund University, Lund, Sweden
[31] Biodiversity Research Institute (CSIC-Oviedo University-Principality of Asturias), University of Oviedo, Campus of Mieres, Mieres, Asturias, Spain
[32] Natural History Museum, University of Oslo, Oslo, Norway
[33] Swedish Museum of Natural History, Stockholm, Sweden
[34] School of Life Sciences, The Chinese University of Hong Kong, Shatin, New Territories, Hong Kong SAR, China
[35] Institute of Zoology, Chinese Academy of Sciences, Beijing 100101, China
[36] State Key Laboratory of Biocontrol, School of Ecology, Sun Yat-sen University, Shenzhen 518107, China
[37] Cornell Lab of Ornithology, Cornell University, Ithaca, USA
[38] Department of Zoology, National Museum of Nature and Science, Tokyo, Japan
[39] Wildfowl and Wetlands Trust, Slimbridge, United Kingdom
[40] Center of Excellence for Omics-Driven Computational Biodiscovery (COMBio), AIMST University, Kedah, Malaysia
[41] Senckenberg Natural History Collections Dresden, Dresden, Germany
[42] Museo di Geologia e Paleontologia, Dipartimento di Scienze della Terra, University of Torino, Italy
[43] Department of Biology, University of Copenhagen, Copenhagen, Denmark
[44] Department of Vertebrates, Natural Science Museum of Barcelona, Barcelona, Spain
[45] Center for Global Mountain Biodiversity, Globe Institute, University of Copenhagen, Copenhagen, Denmark
[46] Department of Biology, University of Southern Denmark, 5230 Odense M, Denmark
[47] Bündnis Naturschutz in Dithmarschen e.V., Hemmingstedt, Germany

[48] Natural History Museum Bern, Bern, Switzerland
[49] Institute of Ecology and Evolution, University of Bern, Bern, Switzerland
[50]Laboratory of Neurogenetics of Language, The Rockefeller University, New York, NY, USA
[51] Department of Life Sciences, Milner Centre for Evolution, University of Bath, Bath, United Kingdom
[52] Zoological Institute of Russian Academy of Sciences, St. Petersburg, Russia
[53] Center of Natural History, Universität Hamburg, Germany
[54] Zoological Museum of Lomonosov Moscow State University, Moscow, Russia
[55] Department of Biology, University of Trnava, Trnava, Slovakia
[56] Instituto de Ecología, Universidad Nacional Autónoma de México, Ciudad de México, México
[57] Sudurnes Science and Learning Center, Sandgerði, Iceland
[58] Institute of Pharmacy and Molecular Biotechnology, Heidelberg University, Heidelberg, Germany
[59] University of Alaska Museum, Fairbanks, USA
[60] Department of Zoology and Physiology, University of Wyoming, Laramie, WY
[61] Université Grenoble Alpes, Université Savoie Mont Blanc, CNRS, LECA,Grenoble, France
[62] Groupe de Recherche en Ecologie Arctique (GREA), Francheville, France
[63] Institute of Tropical Biodiversity and Sustainable Development, University Malaysia Terengganu, Malaysia
[64] College of Life Sciences, Beijing Normal University, Beijing 100875, China
[65] Department of General Surgery of Sir Run Run Shaw Hospital, Zhejiang University School of Medicine, Hangzhou 310016, China

*contributed equally
# Correspondence address. Peter A. Hosner, Natural History Museum of Denmark, University of Copenhagen, Copenhagen, Denmark. E-mail: peter.hosner@snm.ku.dk; and Shaohong Feng, Center for Evolutionary and Organismal Biology, Liangzhu Laboratory, Zhejiang University School of Medicine, Hangzhou 310058, China. E-mail: .fengshaohong@zju.edu.cn

ORCID:
Peter A. Hosner[0000-0001-7499-6224]

Abstract: Background: Reference genome assemblies are essential infrastructure for investigating phylogeny, and population/conservation genetics of wild organisms. Birds serve as model vertebrates in ecology and evolutionary biology due to their well-documented natural histories and extensive community science data. Findings: We release a set of 350 newly assembled avian genomes, which, when combined with 97 previously published genomes, represent 447 of the bird species recorded in Denmark, the Faroe Islands, and Greenland— the largest regional dataset of a vertebrate group to date. These genomes are published for various research activities. Conclusions: This data release advances the global effort to build comprehensive and accessible biodiversity genomic resources for the research community.

Keywords: arctic biodiversity, avian genomics, comparative genomics, conservation genetics, evolutionary biology, ecological genomics, wildlife conservation

Introduction

Sequencing and public accessibility of eukaryotic genomes is emerging as a basic need for contemporary and collaborative biological research [1,2]. These primary datasets provide a foundation for addressing fundamental research questions related to the ecology and evolutionary history of life's diversity, and the genetic basis of its morphological traits, health, behavior, and other phenotypes. Genomes also provide essential tools for addressing pressing global challenges, including wildlife conservation and adaptation to global change [3].

In light of this need, the B10K avian genome sequencing consortium was founded in 2010 to organize, generate, and release a standardized set of de novo sequenced draft avian genomes of selected species [4]. Previous data releases from the B10K comprise a set of 48 avian genomes in 2014, representing most avian orders [5–7], followed by an additional 267 new genomes in 2020, representing 218 avian families [8]. To complement these data, here we report the release of an additional 350 avian genomes, which, when combined with 97 relevant previously published avian genomes, totals 447 species that largely represent the natural avian species diversity recorded in Denmark, the Faroe Islands and Greenland. Our rationale for producing this dataset was based on the need to generate comprehensive reference datasets that can be used by national authorities and researchers to assist in conservation-related research. As such, to the best of our knowledge, this data release constitutes the first comprehensive genome dataset of a major vertebrate taxonomic group for any group of nations. These bird species are generally wide-ranging, hence the data will be broadly relevant for researchers with genomic interests working in Scandinavia, Europe, the Western Palaearctic, and Arctic regions as a whole.

Methods

Tissue sample collection and preparation

Tissue samples representing the target species were sourced from museums, research labs, and other biological collections and their associated researchers (Table S1). Species names follow The Howard and Moore Complete Checklist of the Birds of the World, version 4.0, the taxonomy authority used by the B10K consortium. We aimed to be as comprehensive in our catalog as possible, including species that have been extirpated from parts of their former distributions or that occur occasionally or rarely as migrants or vagrants. Hence, not all the samples processed were originally collected in Denmark, Greenland, and the Faroe Islands, although we prioritized the sampling of local subspecies and populations when possible.

Spatial distribution of focal species

To map the utility of the genome set across the Western Palearctic region, we used observation records of the 447 focal bird species obtained from the Global Biodiversity Information Facility (GBIF) to model their geographical distributions. Species distribution models were constructed with the R package 'ENMeval' [9] for translating the observational records into spatial ranges. To avoid model overfitting, we only retained 7 of the 19 bioclimatic variables used to construct the models [10]. Each of the 447 individual range models was then overlaid to create a composite map (Figure S1).

DNA extraction

Genomic DNA was extracted from blood and tissue samples using the ‘MagMAX DNA Multi-Sample Ultra 2.0` kit (Thermo Fisher) following the manufacturer’s guidelines. Post-lysis, we included an RNase A step at 37ºC, followed by bead-based purification and automated isolation on the Kingfisher Duo Prime using the MMX_Ultra2_Cell_Tissue_96_Duo program. Quality control was performed on the 2200 Tapestation System, and all DNA extractions were stored at -80ºC until library construction.

Genome sequencing

Sequencing technologies employed

Multiple library construction and sequencing strategies were used to produce raw reads for newly collected samples. The differences in library preparation methods and sequencing platforms used reflect techniques available at the time samples were processed. For 336 samples, MGI’s single tube Long Fragment Read (st-LFR) technology [11] was used, after which the resulting DNA Nanoballs (DNBTMs) were sequenced with 100 bp paired-end reads (PE100 + 100 + multiple barcode length modes). For eight samples, the 10X Genomics linked-reads technology was used, followed by sequencing on BGIseq-500 with 150 bp paired-end reads (PE150 + 100 + 42 barcode read length). For six samples, standard Next-Generation technology was used to construct small insert size libraries (300-500 bp), which then underwent DNA Nanoball (DNBTM) generation and DNBSeq sequencing with 150 bp paired-end reads (PE150 + 150 + 10 barcode read length). In total, we produced ~60 Tb of sequencing data for 350 newly released Danish bird species. With an average genome size of 1.2 Gb, the approximate average genome coverage in our dataset is 143 x.

Bioinformatics analysis

Assembly of genomes

For the 336 samples sequenced using st-LFR technology, data were initially transformed into 10X Genomics linked-reads format. Subsequently, this sample set, together with eight samples sequenced by 10X Genomics linked-reads technology, were introduced into the Supernova software (v2.0.1) [13] to assemble the genomes under the "pseudohap" mode. After removing scaffolds with "N" > 80%, gap filling was also done with Gapcloser (v1.12) [12] and the paired-end information as above.

For the six samples sequenced by the standard Next-Generation technology, clean reads were assembled using the SOAPdenovo (v2.04) [12] with a K-mer size of 23-mer following the B10K family-phased assembly strategy [8]. After removing scaffolds with "N" > 80%, GapCloser (v1.12) [12] was used to close the intra-scaffold gaps based on the paired-end information.

Gene structures annotation

Annotation of protein-coding genes was conducted with a homology-based method for both the 350 newly sequenced and 97 published Danish bird species following the pipeline implemented by the B10K consortium [8]. The reference gene set consists of the primary reference gene set (20,194 genes), the supplemental human gene set (20,169 genes), and the supplemental transcriptome gene set (5,257 transcripts). The protein sequences in the primary reference gene set were first aligned to each genome using TBLASTN (v2.2.26) [15] with an e-value cut-off 1e-5, and multiple adjacent hits of the same query were connected by genBlastA (v1.0.4) [16]. Homologous blocks with a length greater than 30% of the query protein length were retained. Each connected hit region was later extended to include its 2 kb upstream and downstream flanking regions, on which gene structure was predicted by Genewise (v2.4.1) [17]. MUSCLE (v3.8.31) [18] was then used to align the annotated protein against its reference protein. Predicted proteins with length ≥ 30 amino acids and an identity value ≥ 40% were retained. Pseudogenes (annotated genes containing > 2 frameshifts or > 1 premature stop codon) and retrogenes were further removed. Next, gene models that overlapped in > 40% of their coding sequence were clustered into one group and the one with the highest identity to the reference proteins was retained to form a non-redundant gene set for each species. Two supplemental gene sets were also used for homology-based gene prediction for these newly released assemblies as above, but only the newly annotated loci from these supplemental sets were kept. Finally, all candidate annotated genes that had > 10 duplications, or were single-exon genes yet contained > 70% repeat sequences in the coding region, were removed.

Repeats annotation

Tandem repeats were identified by Tandem Repeat Finder (v4.09) [19], and transposable elements (TEs) were annotated using a combination of homology-based RepeatMasker (v4.1.2), and de novo methods with RepeatModeler (v2.0.2a) [20] and LTR_Finder (v1.07) [20,21]. The homology-based annotation of TEs was performed by RepeatMasker with parameters "-nolow -no_is -norna -engine ncbi -parallel 1") at the DNA level with its built-in library. The de novo repeat annotation of all 447 species was done by RepeatModeler and LTR_Finder with default parameters to first build a de novo repeat library for each assembly, which was further used by RepeatMasker to predict repeats for each species. The *Ficedula albicollis* (NCBI:txid59894) genome (GCA_000247815.2) was omitted due to an undeterminable error during the annotation of repeats, and the other genomes were sufficient for comparison.

Overview of 447 genomes

Species diversity

We obtained fresh tissue samples for all 497 species recorded in Denmark and all breeding birds of the Faroe Islands and Greenland, save two extinct species, when we ceased sample collection in 2023 (Supplementary Figure 1). However, not all extractions were successful in isolating high molecular-weight DNA. When samples failed to yield adequate DNA quality and quantity, we sought replacement samples. Yet for some species, no replacement was found, or obtaining and sequencing a replacement would have delayed the data release substantially.

The total genome dataset therefore comprises 447 species. This includes the vast majority of the 497 bird species recorded in Denmark up to 2003 (BirdLife Denmark). It also includes most regular breeding species and regular visitors to Greenland and the Faroe Islands. The majority of the missing species (30) are vagrants, rare visitors, and migrants that have only been sporadically recorded in Denmark. Twenty of the missing species are regularly occurring. Additionally, the dataset reflects avian taxonomic diversity well. All bird orders and families occurring in the target regions are represented.

Genome statistics

The genome assemblies of the 350 newly released avian species ranged from 1.0 to 1.4 Gb, with average scaffold N50 = 8.2 Mb, and contig N50 = 105.3 kb (Figure 1, Supplementary Figure 2-4). Only 6.4% of the core genes in aves_odb10 could not be predicted on these newly released genomes (ranging from 2.2% to 31.5%), suggesting that the completeness of these genomes was suitable for most comparative genome analyses (Figure 2, Supplementary Figure

5). An average of 89.1% complete core genes in the newly released genomes was comparable to that of the previously published genomes in the dataset (90.4%), but slightly less than bird genomes produced to VGP standards (92–95%, [22]). Duplication rates inferred from the BUSCO measurements varied between 0% and 10.1% among the genomes. On average, only 4.6% of the core genes were partly annotated for the 350 new genomes. Using the homologous annotation method, the 447 genomes were predicted to contain an average of 15,915 protein-coding genes (ranging from *Clangula hyemalis*: 11,235 to *Chroicocephalus ridibundus*: 20,269), similar to previously published bird genomes [8]. The average gene length and coding sequence length are ~154.6 and 1.3 kb, respectively. Genes contained on average ~7 exons, with an average length of 173 bp and an average intron length of 2.2 kb (Figure 3, Supplementary Figures 6-8). Consistent with the results of previous studies ([5,8]), the genomes of these newly sequenced species contained a low proportion of transposable elements (11.9% on average), except for 8 outliers (all species of woodpeckers in Piciformes; Figure 4, Supplementary Figure 9). While nearly all analyzed genomes exhibited similar repeat content, they varied in content for each category. For all species, the most abundant repeat category was LINE elements (7.2% on average). Northern Wryneck (*Jynx torquilla*) has the richest LINE repeats of 27.4%, while Yellow-nosed Albatross (*Thalassarche chlororhynchos*) has the richest SINE repeats of 0.23%. Lapland Longspur (*Calcarius lapponicus*) had the most genome sequences identified as LTR (10.4%). Of the newly released genomes, 53% are from ZW females, and 47% are from ZZ males; the latter lack draft W chromosome scaffolds.

Data Validation and Quality Control

Sample verification

Voucher specimens were verified against reference collections. Mitogenomes were BLAST searched against references in the NCBI database. If no reference mitogenome was available, we checked NCBI for commonly sequenced mitochondrial genes (COI, ND2, CYB) as a reference to verify sequence authenticity. For genomes that lacked a mitochondrial assembly, which was frequent when blood was the source tissue, we used a collection of nuclear markers well-represented on NCBI for birds (FGB, GAPDH, MB, MUSK, ODC, RAG1, TGFb2).

Genome sequencing

Quality control steps for raw reads before assembly using the SOAPfilter2 package (v2.2) [12] were:

1) Remove reads with more than 10% of N bases
2) Remove reads with more than 40% low-quality bases (Phred score < = 10)

3) Remove reads with undersized insert size
4) Filter out the PCR duplicates

Genome Assembly

Assembly quality of both the 350 newly sequenced and the 97 previously published species was assessed by contig N50, scaffold N50, and total assembly length. Genome completeness was measured with BUSCO (v5.4.6) [14] using aves_odb10 as the reference gene set for these species. Three standard categories of BUSCO results were assessed as follows:

1) Complete and single-copy BUSCOs (S)
2) Complete and duplicated BUSCOs (D)
3) Fragmented BUSCOs (F)

The complete (S and D) and fragmentary (F) hits were combined to assess the degree of genome completeness. Only 24 species had genomes with <80% completeness, the lowest being 68.5%. For the six short insert library species, the BUSCO completeness was lower, and percentages ranged from 79.3% to 90.8%.

Reuse Potential

We created this dataset to support future research on birds, especially those important to the biodiversity of Denmark, Greenland, and the Faroe Islands. While it will be useful for broad avian evolutionary studies, we expect its most lasting value will be in conservation genetics and management of the species included. Potential uses include population genomics, mapping genetic structure and gene flow, identifying hybrid zones, studying local adaptation, and investigating disease ecology.

As sequencing technologies continue to improve (and associated costs decrease), the quality of genomes that can be sequenced and de novo assembled continues to improve. The quality of the genomes released here represents a level that was feasible at the time of this project, given the financial, technical, and biosample constraints available to the project. A future aim is to continue improving quality, for example, by incorporating additional technologies, such as long reads and scaffolding tools like Hi-C, as implemented in initiatives such as the Vertebrate Genome and Darwin Tree of Life Projects [22]. Nevertheless, we believe the quality will be suitable for many of the above possible end uses.

Conclusion

A key challenge in biodiversity genomics is obtaining tissue samples with high-quality, high molecular-weight DNA. For this project, we prepared libraries from samples with DNA fragments longer than 10,000 bp. However, many libraries with fragments of 10,000–20,000 bp failed to produce genome assemblies of acceptable quality, and of those that did, benchmark BUSCO scores suffered. For stLFR sequencing, DNA fragments over 60,000 bp are ideal, yet many of our samples—particularly from salvaged birds found dead (e.g., collision victims)—fell short of this standard. These salvaged specimens had a success rate of about 50%, while freshly collected museum specimens and blood samples had success rates exceeding 90%. While salvaged specimens are valuable for collection building and short-read sequencing, their average poorer DNA quality limits their usefulness for de novo genome assemblies, and they should be considered as a secondary option.

Although our release of avian genomes is comprehensive, it is not complete. When completing the project, we made practical decisions about the final dataset because the genomes of some relatively common species produced substandard assemblies. We flag notable absences in our dataset from the Danish avifauna and highlight alternative genomic resources, including Garganey (*Spatula querquedula*; Iridian genomes GCA_032191525.1; NCBI:txid75856), Black-necked Grebe (*Podiceps nigricollis* in prep; DTOL; NCBI:txid85099), Wood Pigeon (*Columba palumbus*; DTOL GCA_965250375.1; NCBI:txid8934), Black-throated Loon (*Gavia arctica*; NCBI:txid57069), European Herring Gull (*Larus argentatus;* DTOL GCA_964417175.1; NCBI:txid35669), Short-eared Owl (*Asio flammeus*; NCBI:txid56267), Sand Martin/Bank Swallow (*Riparia riparia*; Institute of Ecology and Evolution University of Bern, GCA_020917445.1; NCBI:txid88110), Black Redstart (*Phoenicurus ochruros,* Kent State University GCA_050234705.1; NCBI:txid358815), and Yellowhammer (*Emberiza citrinella*; in prep, DTOL; NCBI:txid37595). In addition, one Faroese breeder, European Shag (*Phalacrocorax aristotelis*; DTOL GCA_949628215.1; NCBI:txid126867), and one Greenlandic breeder, Snow Goose (*Anser caerulescens*; Iridian Genomes GCA_051253075.1; NCBI:txid8849), remain targets for future work. Finally, during manuscript preparation, we identified that our genome of the Common Moorhen (*Gallinula chloropus*; NCBI:txid9123) pertains to the Common Gallinule (*G. galeata*; NCBI:txid1109398), following a recent taxonomic change. However, we note that a true *G. chloropus* assembly is available from the Sanger Institute's Darwin Tree of Life Project GCA_964237585.1. We intend to identify additional high-quality samples to fill these gaps as the B10K consortium continues its aims.

Availability of Source Code and Requirements

Project name: DanishBirdsGenome
Project home page: https://github.com/ChenGuangji/DanishBirdsGenome
License: MIT license

Operating system(s): Cross platform
Programming language: Python, Perl, R, Unix shell
Genome assembly pipeline: https://github.com/BGIQingdao/stlfr2supernova_pipeline
Genome annotation pipeline: https://github.com/B10KGenomes/annotation
Statistics and visualizations: https://github.com/ChenGuangji/DanishBirdsGenome

Data Availability

Genome sequencing data, genome assemblies, and annotations of the 350 species generated in this study have been deposited in the European Nucleotide Archive (ENA) under accession ERP190789. The supporting data [23] have been deposited in the GigaScience GigaDB database [24]. Sample information for each genome and genome statistics can also be viewed online at [25]. The study used 97 publicly available assemblies from NCBI with the Genome Accession IDs listed in Supplementary table S1. The reference genomes, gene sets and published RNA-sequencing data used in the gene annotation and alignment construction of this study are available from Ensembl and NCBI. The database used in the transposable elements annotation is available online [26].

Guidelines for data usage and citation

This data release paper and the GenBank accession information should be cited in relevant publications. The B10K consortium is actively working on large-scale, higher-level avian phylogenomic projects incorporating data released herein. We request that authors with similar research interests contact the B10K steering committee rather than opting to pursue potentially competing projects.

Declarations

All field sampling efforts followed appropriate local permission structures, laws, and regulations, including the Nagoya Protocol and Institutional Review Boards where they apply. All tissue samples were received and processed at Globe Institute, University of Copenhagen, which is certified to receive and process biological tissues under a permit for Animal By-Products DK-123-oth-905637 issued by The Danish Veterinary and Food Administration. CITES samples received from outside the European Union are under the CITES Scientific Exchange Exemption, registration number DK 014 issued by the Danish Environmental Protection Agency.

Competing interests

The authors declare no competing interests.

Funding

The authors would like to acknowledge the following sources of funding: Villum Foundation Villum Investigator grant no. 25900 to GZ; Villum Foundation Villum Investigator grant (no. 25925 CR; Danish National Research Foundation DNRF143 to MTPG; National Key Research and Development Program of China grant no. 2023YFA1800500 and National Natural Science Foundation of China grant no. 32170626 to SF, Independent Research Fund Denmark no. 1054-00039B to PAH; Spanish Ministry of Science, Innovation and Universities, and the European Regional Development Fund PID2022-140091NB-I00 to JCI.

Authors' contribution

Conceptualization: EJ, CR, GRGr, MTPG, FL, GZ, SF, and PAH conceived of the study. Data Curation: MJA, VA, RA, SA, SMB, RTB, VB, AC, TMB, SB, WC, SC, WD, SVE, MMF, JF, JFi, GF, JFu, GRGa, LG, GRGr, GG, TH, AH, OH, JCI, LEJ, AJ, USJ, KAJ, WJ, VJ, TK, ADL, FL, JTL, ML, FLi, YL, IJL, IN, MFO, MO, MP, MPa, JQ, AR, ARG, MR, VGR, TS, CJS, AS, MS, SS, GS, TSz, IJS, DTT, PST, AT, AOU, SRV, KWa, MW, KWi, CCW, JJW, JLW, GY, NY, ZZ, SW, JX, and PAH collected and archived tissues from fieldwork. Project Administration, Investigation, and Software: DBO, SD, GC, AP, SSTM, JS, BP, GC, XS, ZS, SF, and PAH performed project organization, lab work, and bioinformatics. Writing - Original Draft, Review & Editing: DBO, SD, GC, MTGP, GZ, SF, and PAH drafted the manuscript, which was edited and approved by all authors.

Acknowledgments

The authors would like to thank the various field workers, museums, universities and other genetic resource archives, permitting officials, and other support and administrative staff without whom sample collection programs could not function: Natural History Museum Denmark (NHMD), University of Copenhagen, Smithsonian National Museum of Natural History (USNM), Natural History Museum of Oslo (NHMO), Charles University, Leibniz Institute for Zoo and Wildlife Research (IZW), Swedish Natural History Museum (NMR), Zoological Museum of Moscow University (ZMMU), Museum of Comparative Zoology (MCZ), Museum of South Western Biology (MSB), University of New Mexico, Steinhardt Museum of Natural History (SMNHTAU), Institute of Pharmacy and Molecular Biotechnology (IPMB); Heidelberg University, University of Washington Burke Museum (UWBM), Museum of Natural Science Barcelona (MZB), National Museum of Nature and Science Japan, Cornell University Museum of Vertebrates (CUMV), Barcelona Zoo, University of Bath (UOB), University of Milano, Museo di Geologia e Paleontologia, Università degli Studi di Torino, Universidad de Oviedo (FCO), Muséum national d'Histoire naturelle (MNHM), Institute of avian research (IAR), Wildlife Wetlands Trust Slimbridge (WWT), School of Ecology, Sun Yat-sen University, Shenzhen, University of Hamburg (UHH), Senckenberg Natural History Collections (SMF), Yamashina Institute for Ornithology Japan (YIO), Royal Ontario Museum (ROM), University of Toronto, National Institute for Environmental Studies (NIES), Université Savoie Mont Blanc, Institute of

Zoology Chinese Academy of Sciences (IOZ), University of Alaska Museum (UAM), University of Lethbridge, and Lund University. Zhe Jiang University, Liangzhu Laboratory, and China National GenBank provided computational resources.

References

1. Lewin HA, Robinson GE, Kress WJ, Baker WJ, Coddington J, Crandall KA, et al. Earth BioGenome Project: Sequencing life for the future of life. Proc Natl Acad Sci U S A. 2018;115: 4325–4333. doi:10.1073/pnas.1720115115

2. Lewin HA, Richards S, Lieberman Aiden E, Allende ML, Archibald JM, Bálint M, et al. The Earth BioGenome Project 2020: Starting the clock. Proc Natl Acad Sci U S A. 2022;119. doi:10.1073/pnas.2115635118

3. Formenti G, Theissinger K, Fernandes C, Bista I, Bombarely A, Bleidorn C, et al. The era of reference genomes in conservation genomics. Trends Ecol Evol. 2022. doi:10.1016/j.tree.2021.11.008

4. Zhang G, Jarvis ED, Gilbert MTP. A flock of Genomes. Science. 2014;346: 1308–1309. doi:10.1126/science.346.6215.1308

5. Zhang G, Li C, Li Q, Li B, Larkin DM, Lee C, et al. Comparative genomics reveals insights into avian genome evolution and adaptation. Science. 2014;346: 1311–1320. doi:10.1126/science.1251385

6. Zhang G, Li B, Li C, Gilbert MTP, Jarvis ED, Wang J, et al. Comparative genomic data of the Avian Phylogenomics Project. Gigascience. 2014;3: 26. doi:10.1186/2047-217X-3-26

7. Jarvis ED, Mirarab S, Aberer AJ, Li B, Houde P, Li C, et al. Whole-genome analyses resolve early branches in the tree of life of modern birds. Science. 2014;346: 1320–1331. doi:10.1126/science.1253451

8. Feng S, Stiller J, Deng Y, Armstrong J, Fang Q, Reeve AH, et al. Dense sampling of bird diversity increases power of comparative genomics. Nature. 2020;587: 252–257. doi:10.1038/s41586-020-2873-9

9. Kass JM, Muscarella R, Galante PJ, Bohl CL, Pinilla-Buitrago GE, Boria RA, et al. ENMeval 2.0: Redesigned for customizable and reproducible modeling of species' niches and distributions. Methods Ecol Evol. 2021;12: 1602–1608. doi:10.1111/2041-210X.13628

10. Feng X, Park DS, Liang Y, Pandey R, Papeş M. Collinearity in ecological niche modeling: Confusions and challenges. Ecol Evol. 2019;9: 10365–10376. doi:10.1002/ece3.5555

11. Wang O, Chin R, Cheng X, Wu MKY, Mao Q, Tang J, et al. Efficient and unique cobarcoding of second-generation sequencing reads from long DNA molecules enabling cost-effective and accurate sequencing, haplotyping, and de novo assembly. Genome Res. 2019;29: 798–808. doi:10.1101/gr.245126.118

12. Luo R, Liu B, Xie Y, Li Z, Huang W, Yuan J, et al. SOAPdenovo2: an empirically improved memory-efficient short-read de novo assembler. Gigascience. 2012;1: 18. doi:10.1186/2047-217X-1-18

13. Weisenfeld NI, Kumar V, Shah P, Church DM, Jaffe DB. Direct determination of diploid genome sequences. Genome Res. 2017;27: 757–767. doi:10.1101/gr.213876.116

14. Manni M, Berkeley MR, Seppey M, Simão FA, Zdobnov EM. BUSCO Update: Novel and Streamlined Workflows along with Broader and Deeper Phylogenetic Coverage for Scoring of Eukaryotic, Prokaryotic, and Viral Genomes. Mol Biol Evol. 2021;38: 4647–4654. doi:10.1093/molbev/msab199

15. Altschul SF, Madden TL, Schäffer AA, Zhang J, Zhang Z, Miller W, et al. Gapped BLAST and PSI-BLAST: a new generation of protein database search programs. Nucleic Acids Res. 1997;25: 3389–3402. doi:10.1093/nar/25.17.3389

16. She R, Chu JS-C, Wang K, Pei J, Chen N. GenBlastA: enabling BLAST to identify homologous gene sequences. Genome Res. 2009;19: 143–149. doi:10.1101/gr.082081.108

17. Birney E, Clamp M, Durbin R. GeneWise and Genomewise. Genome Res. 2004;14: 988–995. doi:10.1101/gr.1865504

18. Edgar RC. MUSCLE: multiple sequence alignment with high accuracy and high throughput. Nucleic Acids Res. 2004;32: 1792–1797. doi:10.1093/nar/gkh340

19. Benson G. Tandem repeats finder: a program to analyze DNA sequences. Nucleic Acids Res. 1999;27: 573–580. doi:10.1093/nar/27.2.573

20. Flynn JM, Hubley R, Goubert C, Rosen J, Clark AG, Feschotte C, et al. RepeatModeler2 for automated genomic discovery of transposable element families. Proc Natl Acad Sci U.S.A. 2020;117: 9451–9457. doi:10.1073/pnas.1921046117

21. Xu Z, Wang H. LTR_FINDER: an efficient tool for the prediction of full-length LTR retrotransposons. Nucleic Acids Res. 2007;35: W265–8. doi:10.1093/nar/gkm286

22. Rhie A, McCarthy SA, Fedrigo O, Damas J, Formenti G, Koren S, et al. Towards complete and error-free genome assemblies of all vertebrate species. Nature. 2021;592: 737–746. doi:10.1038/s41586-021-03451-0

23. Øksnebjerg D B; Nielsen S D; Chen G; Andersen M J; Almagro V; Ambrosini R; et al. (2026): Supporting data for "A comprehensive reference genome assembly dataset of birds inhabiting Denmark, Greenland, and the Faroe Islands" GigaScience Database. https://doi.org/10.5524/102824

24. Xiaoqiang Li, Cong Hua, Qian Yue, Zhiyong Li, Jiawei Tong, Ziheng Luo, et al. GigaDB: a redesigned repository for data publishing and management. GigaScience. 2026; 15: giag047. doi:10.1093/gigascience/giag047

25. The B10K Project. https://b10k.com/. Accessed 19 May 2026.

26. RepeatMasker.http://www.repeatmasker.org. Accessed 19 May 2026.

Tables and Figures

Figure 1. Assembly statistics for 350 newly released avian genomes are colored by each of the three sequencing strategies used.

Figure 2. BUSCO evaluation for 350 newly released avian genomes of the birds inhabiting Denmark, Greenland, and the Faroe Islands.

Figure 3. Ten indicators of gene annotation statistics for the 350 newly released avian genomes. The y-axis measures the number of occurrences; box plots, and density distributions show the distribution of values across the genomes.

Figure 4. Transposable element (TE) percentages for the 350 newly released avian genomes. Box plots are shown for orders with at least three sequenced species.

Supplementary Figure 1. Overlapping spatial distribution of the 447 species represented in the dataset, illustrating broad coverage of the Western Palearctic avifauna

Supplementary Figure 2. Assembly statistics for 350 newly released avian genomes.

Supplementary Figure 3. Assembly statistics for all 447 avian genomes, published & new (paper & website.

Supplementary Figure 4. Assembly statistics for all 447 avian genomes.

Supplementary Figure 5. BUSCOs evaluation for all 447 avian genomes.

Supplementary Figure 6. Eight indicators of gene annotation statistics for 350 newly released avian genomes across orders. Box plots are shown for orders with at least three sequenced species.

Supplementary Figure 7. Eight indicators of gene annotation statistics for all 447 avian genomes.

Supplementary Figure 8. Eight gene annotation statistics for all 447 avian genomes across orders. Box plots are shown for orders with at least three sequenced species.

Supplementary Figure 9. Transposable element (TE) percentage for all 447 avian genomes. Box plots are shown for orders with at least three sequenced species.

Figure 1

Click here to access/download;Figure;Figure_1.png

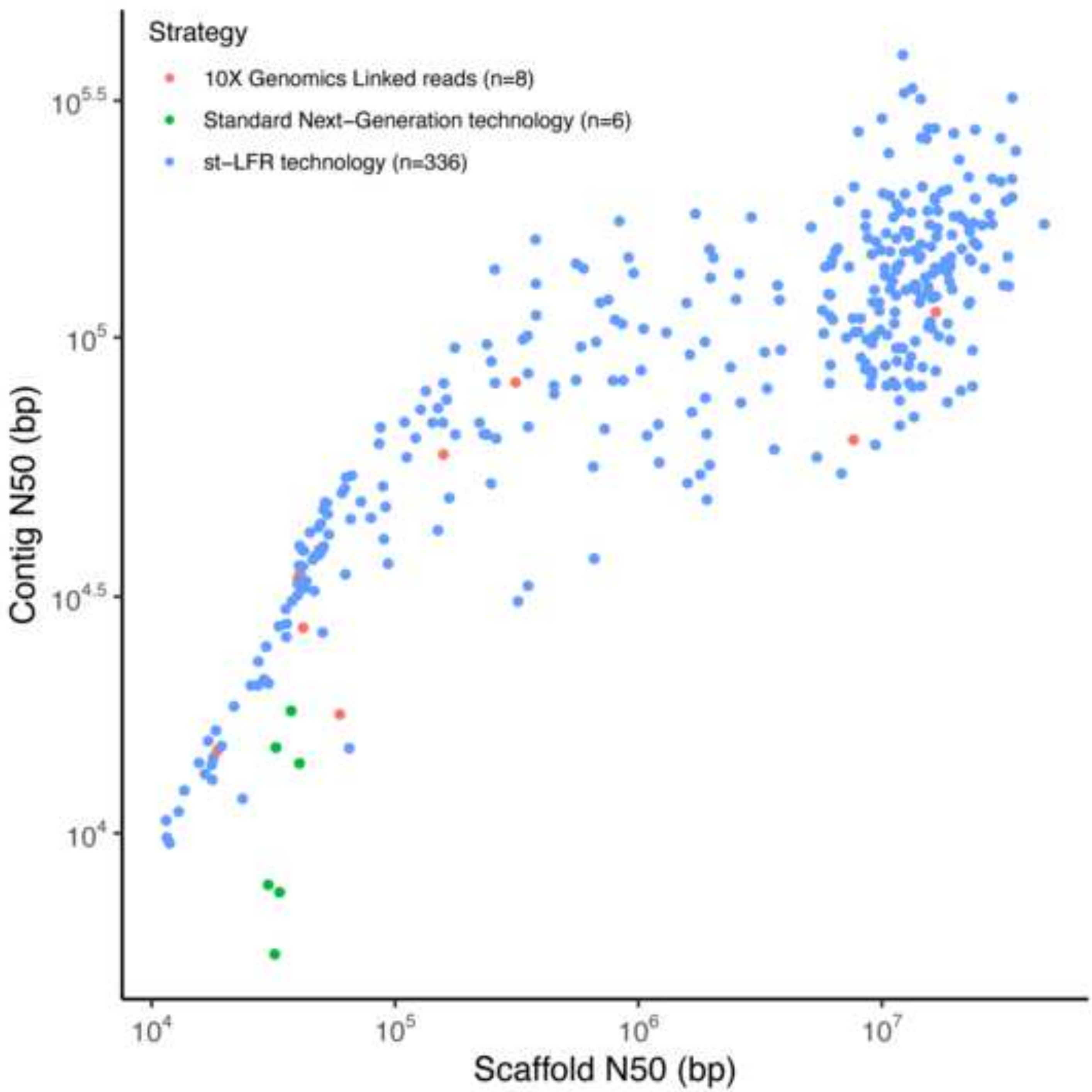

Figure 3

Click here to access/download;Figure;Fig3_20251230_newly_with_published.pdf

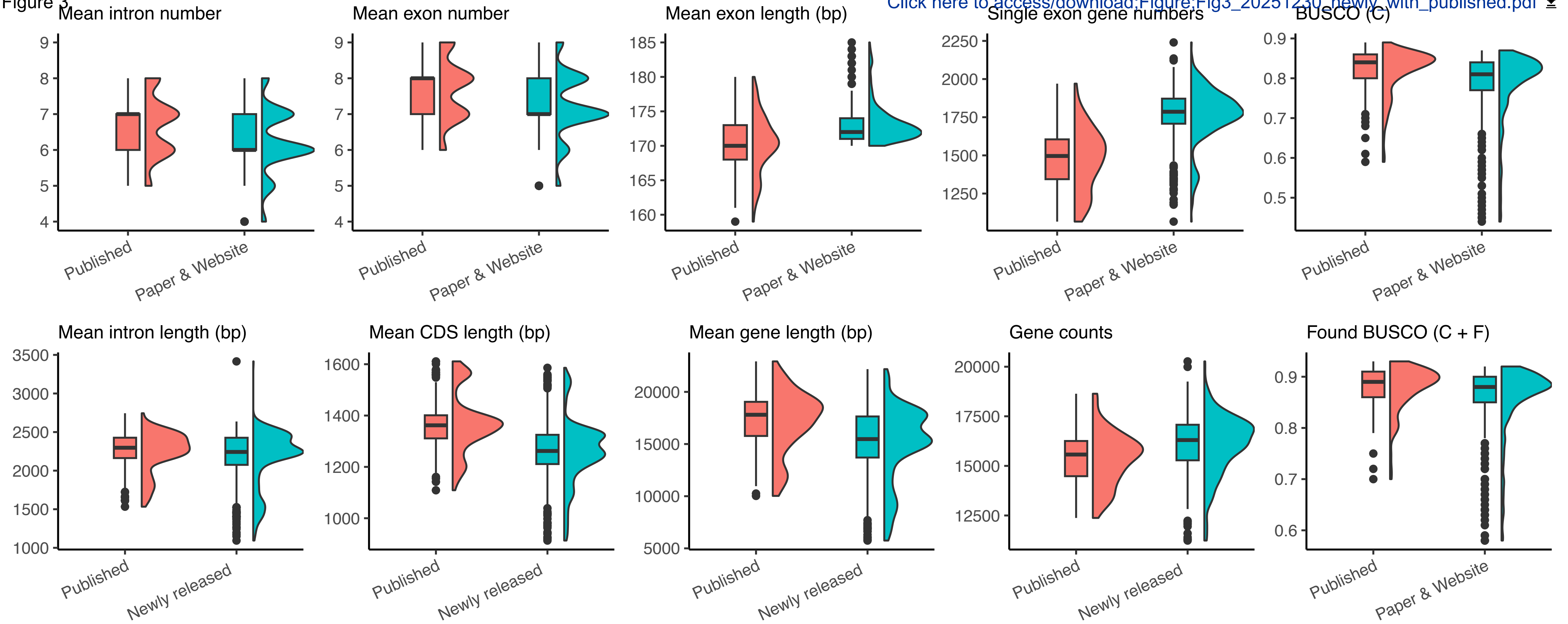

Figure 2

Click here to access/download;Figure;Figure_2.png

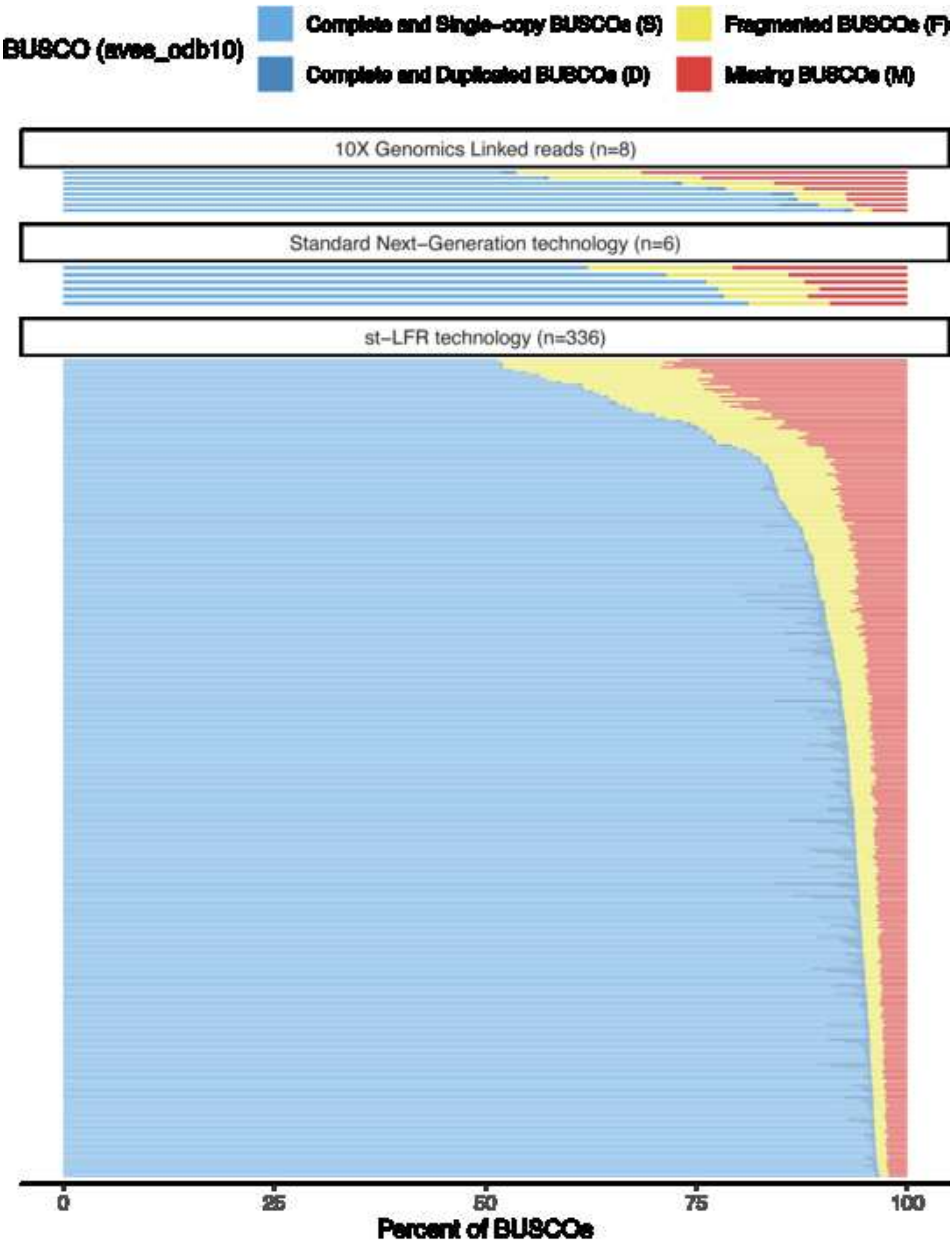

Figure 4

Click here to access/download;Figure;Figure_4.png

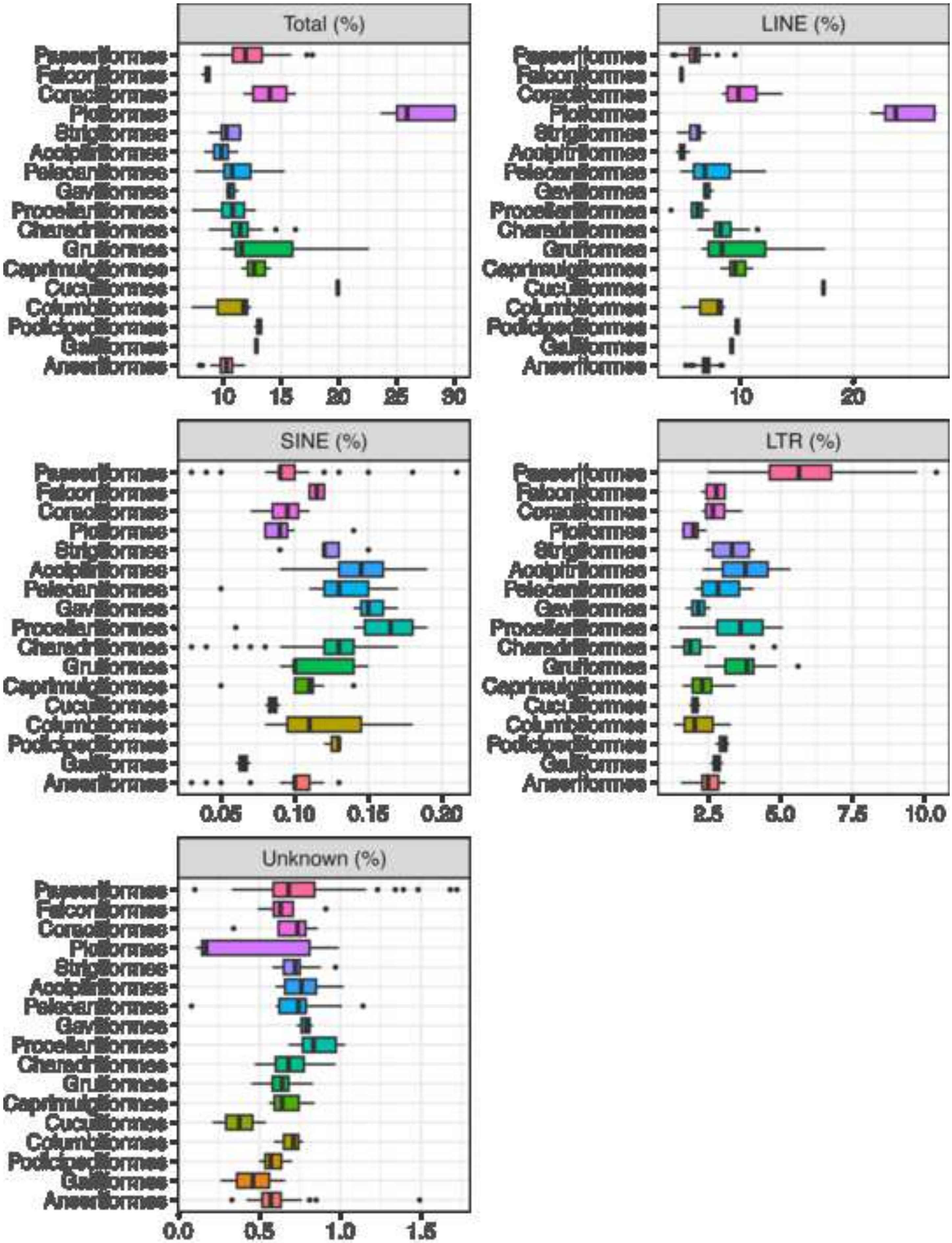

Figure S1

Click here to access/download;Figure;Su

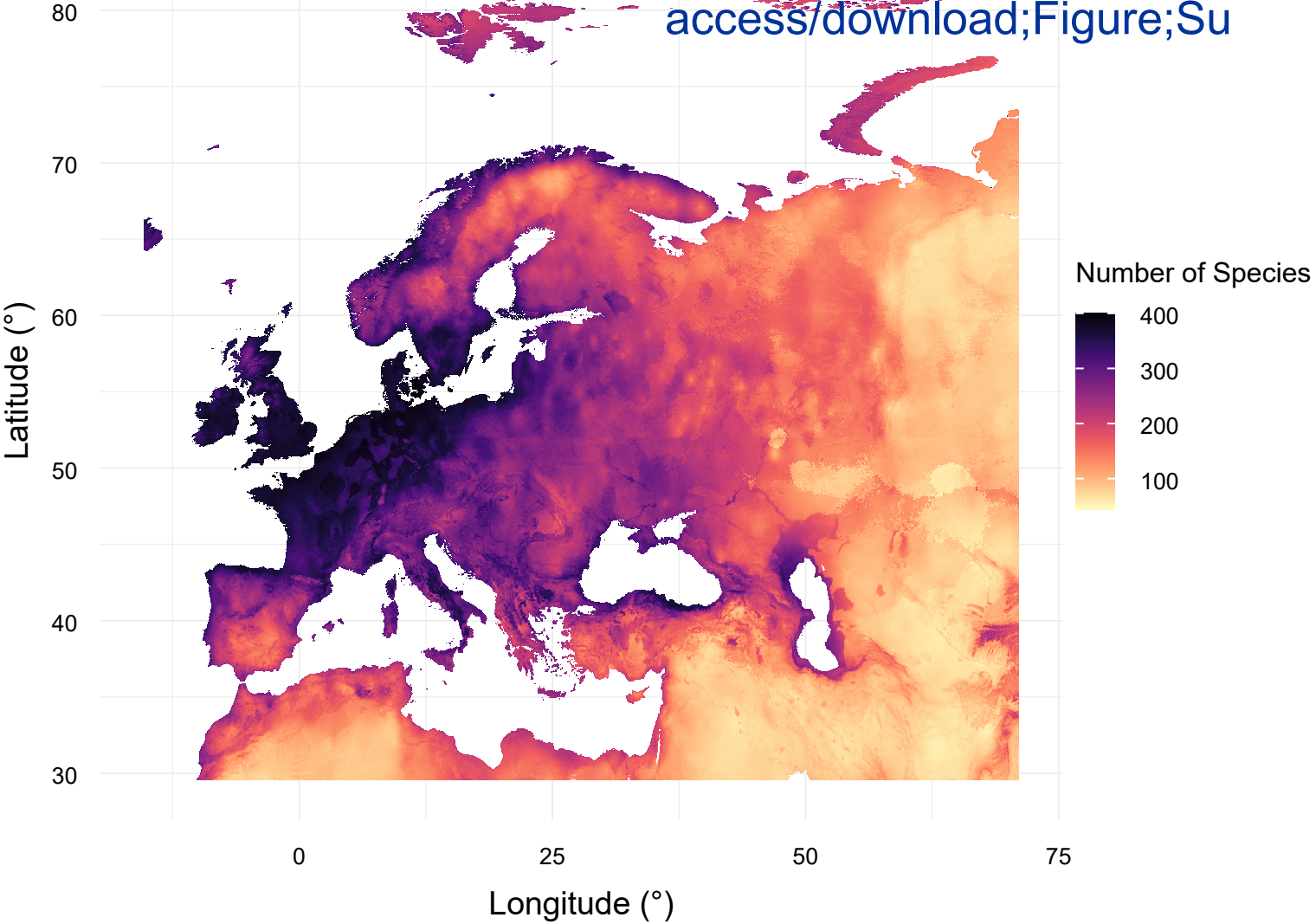

Figure S2

Click here to access/download;Figure;Supplementary Figure 2.png

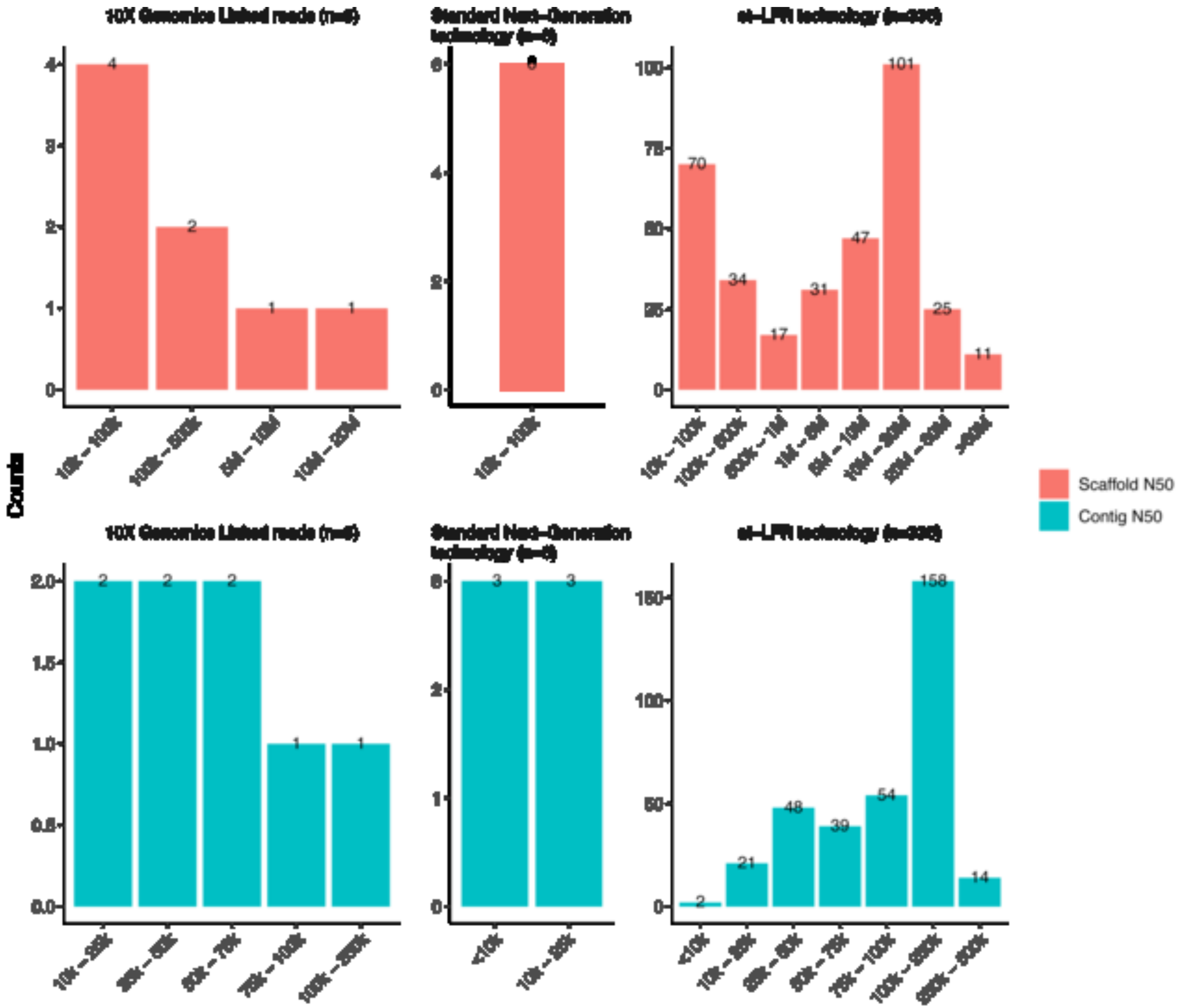

Figure S4

Click here to access/download;Figure;Supplementary Figure 4.png

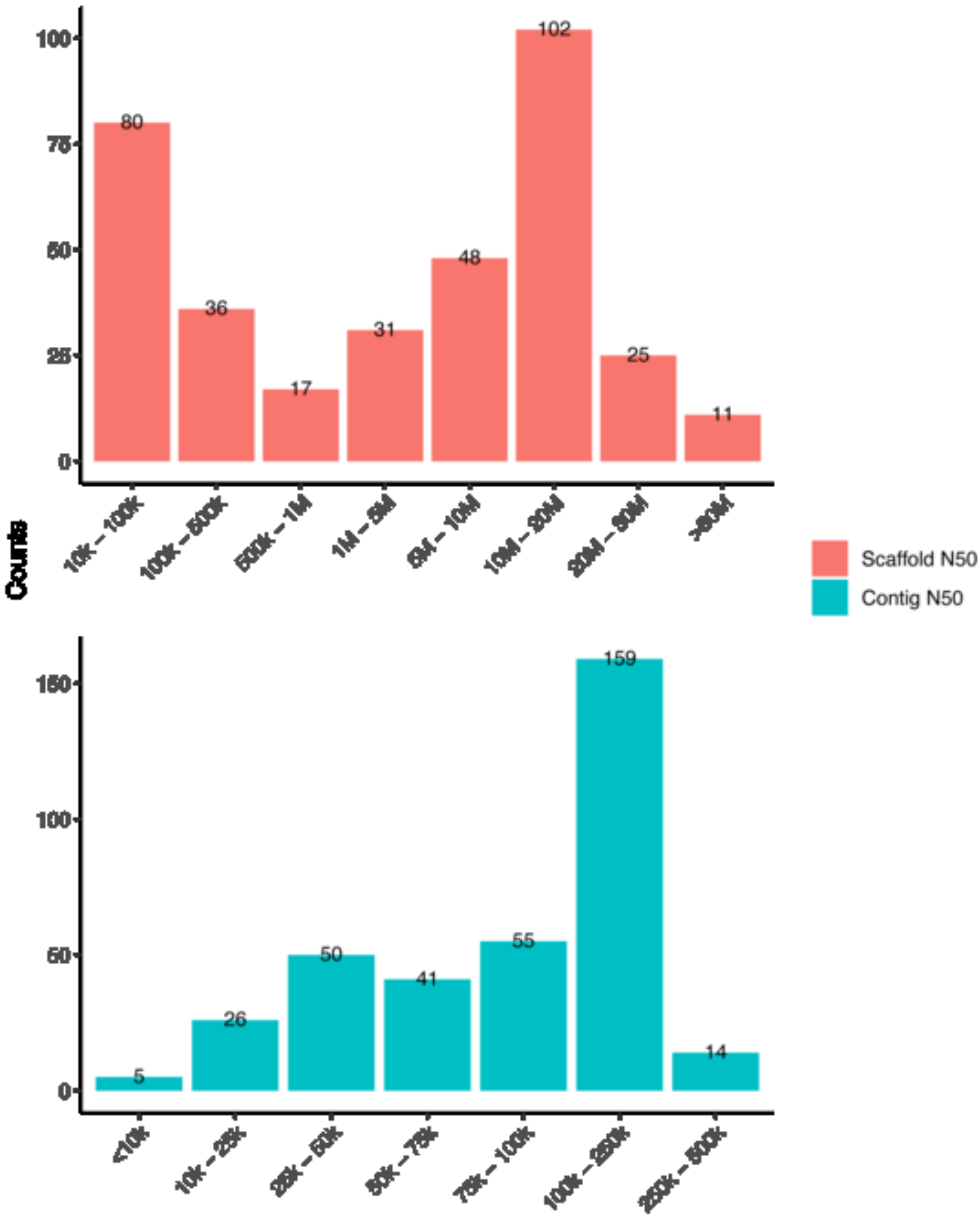

Figure S3

Click here to access/download;Figure;FigS3_20251224_newly_with_published.pdf

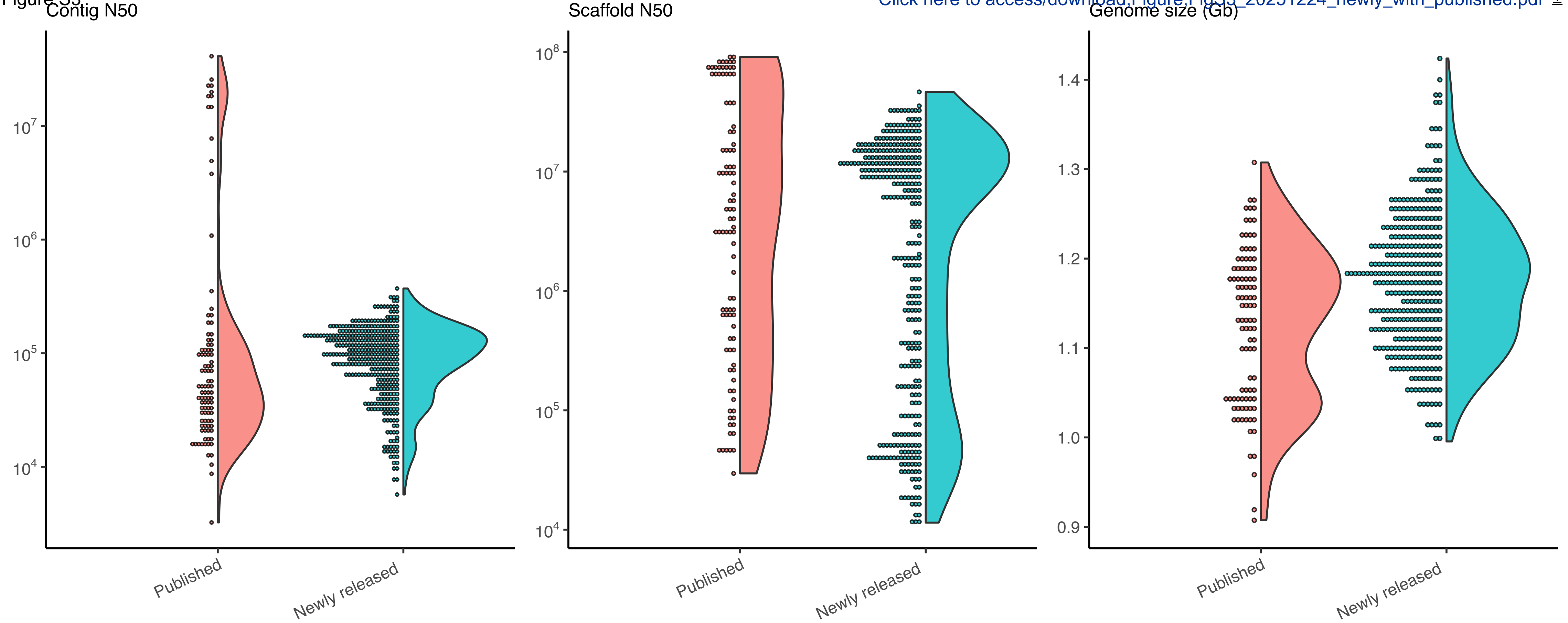

Figure S5

Click here to access/download;Figure;Supplementary Figure 5.png

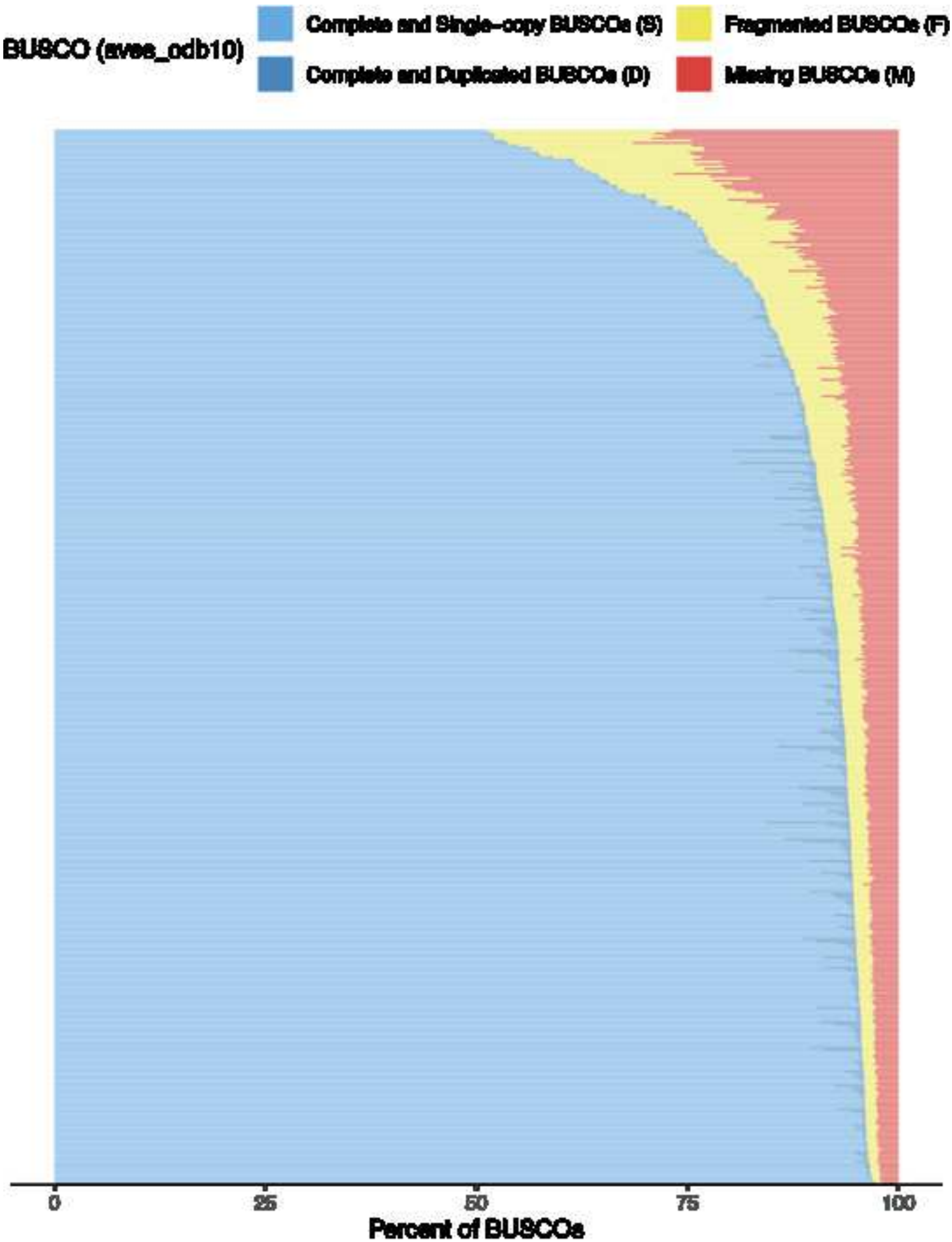

Figure S6

Click here to access/download;Figure;Supplementary Figure 6.png

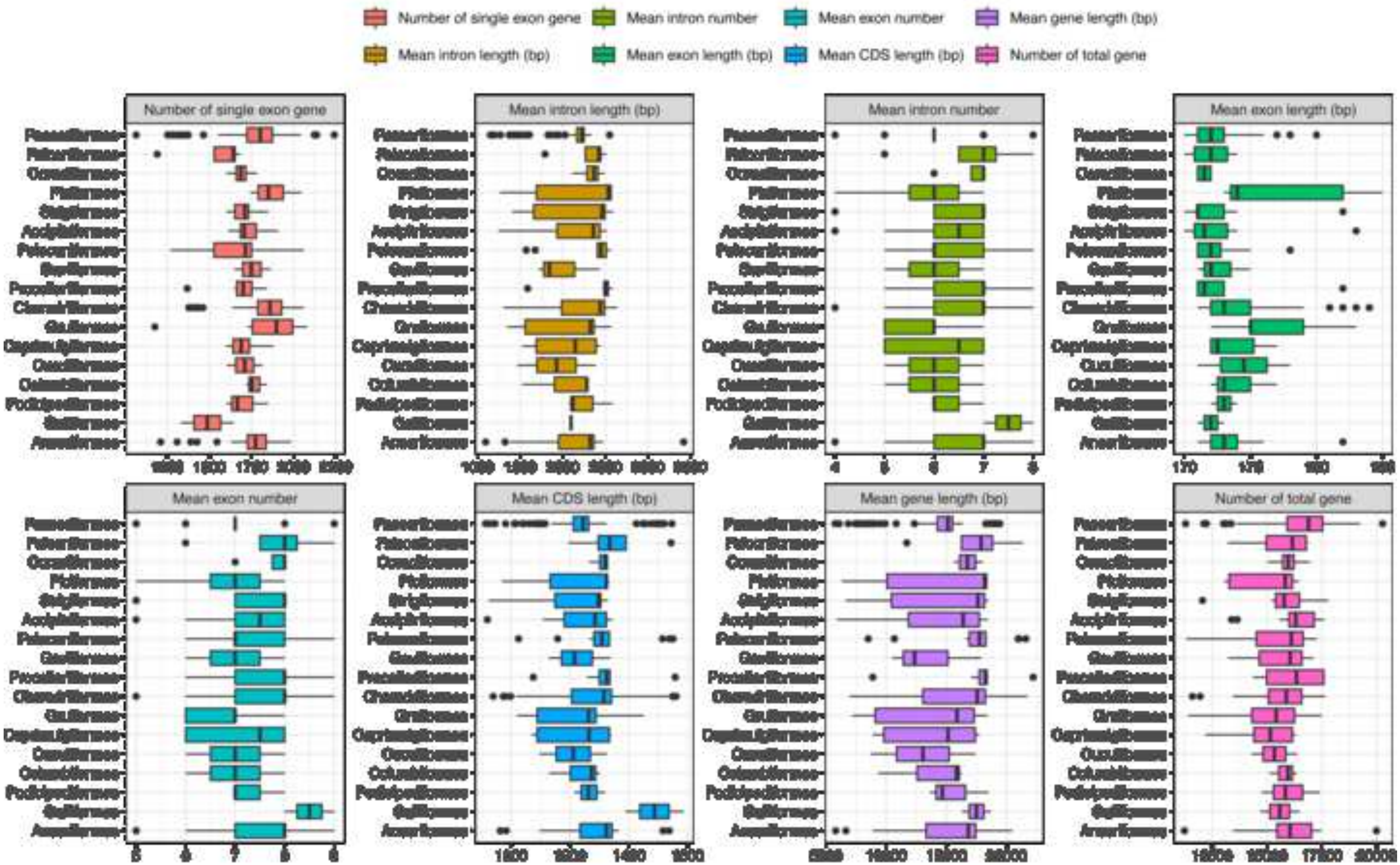

Figure S7

Click here to access/download;Figure;Supplementary Figure 7.png

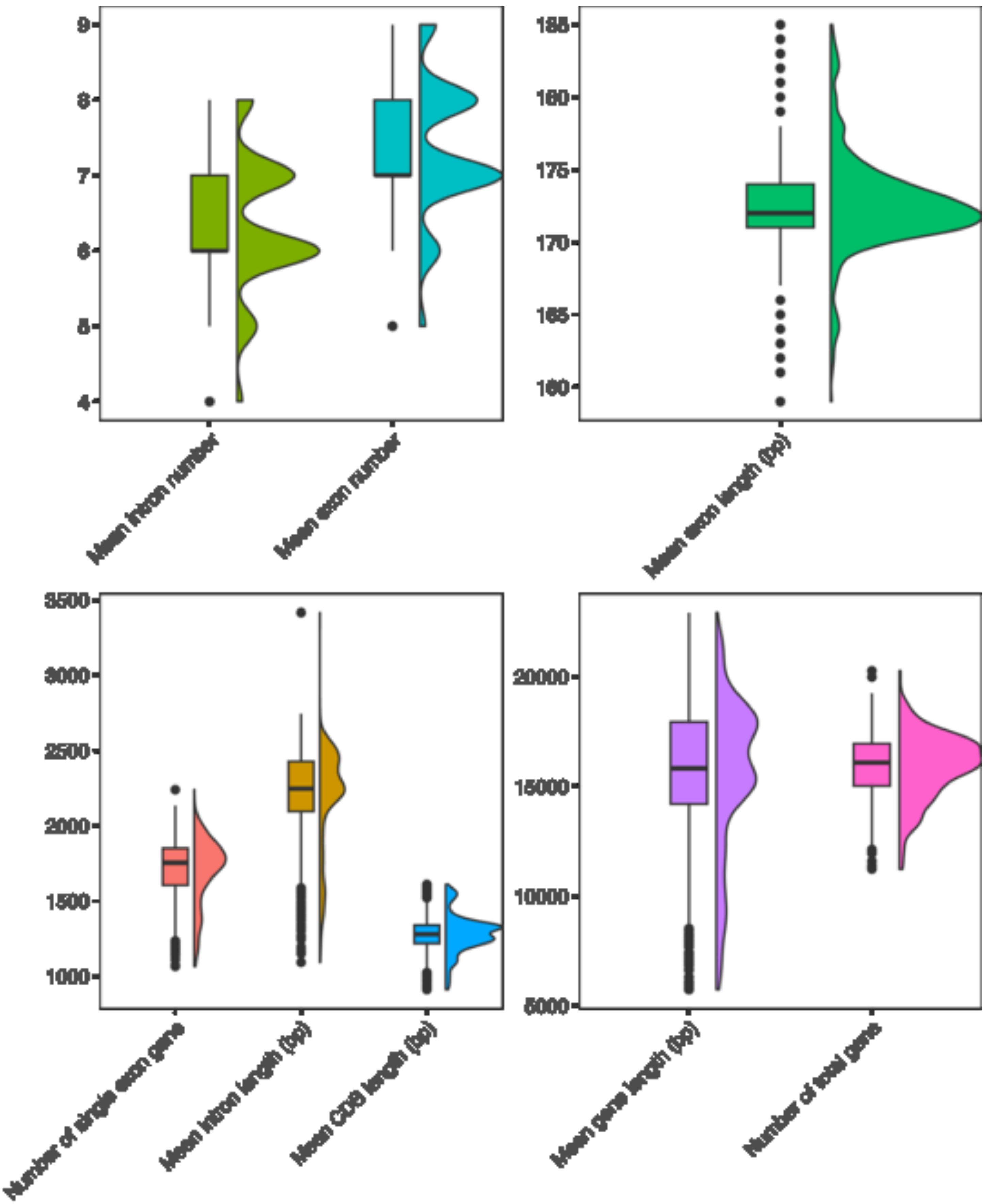

Figure S8

Click here to access/download;Figure;Supplementary Figure 8.png

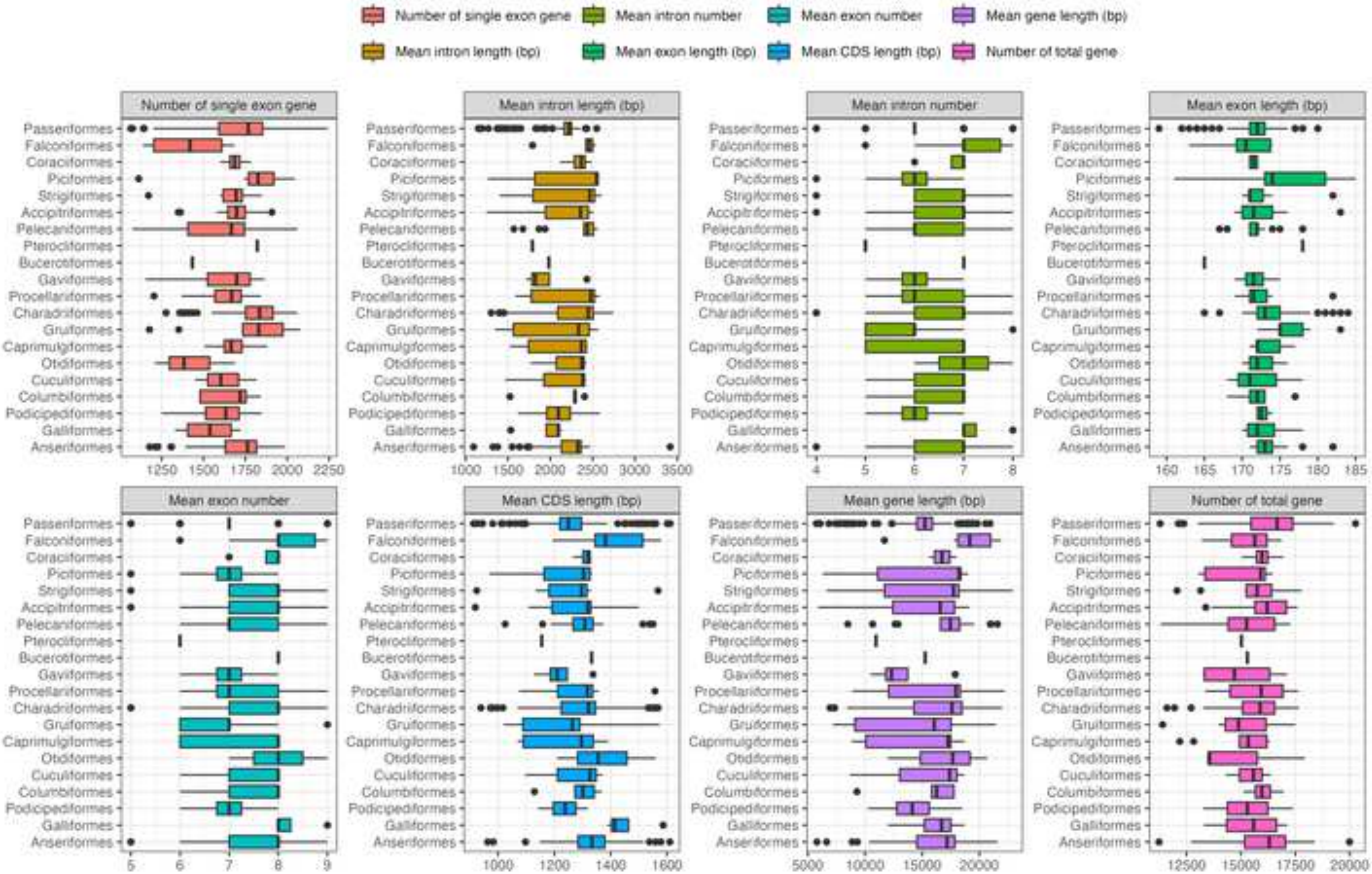

Figure S9

Click here to access/download;Figure;Supplementary Figure 9.png

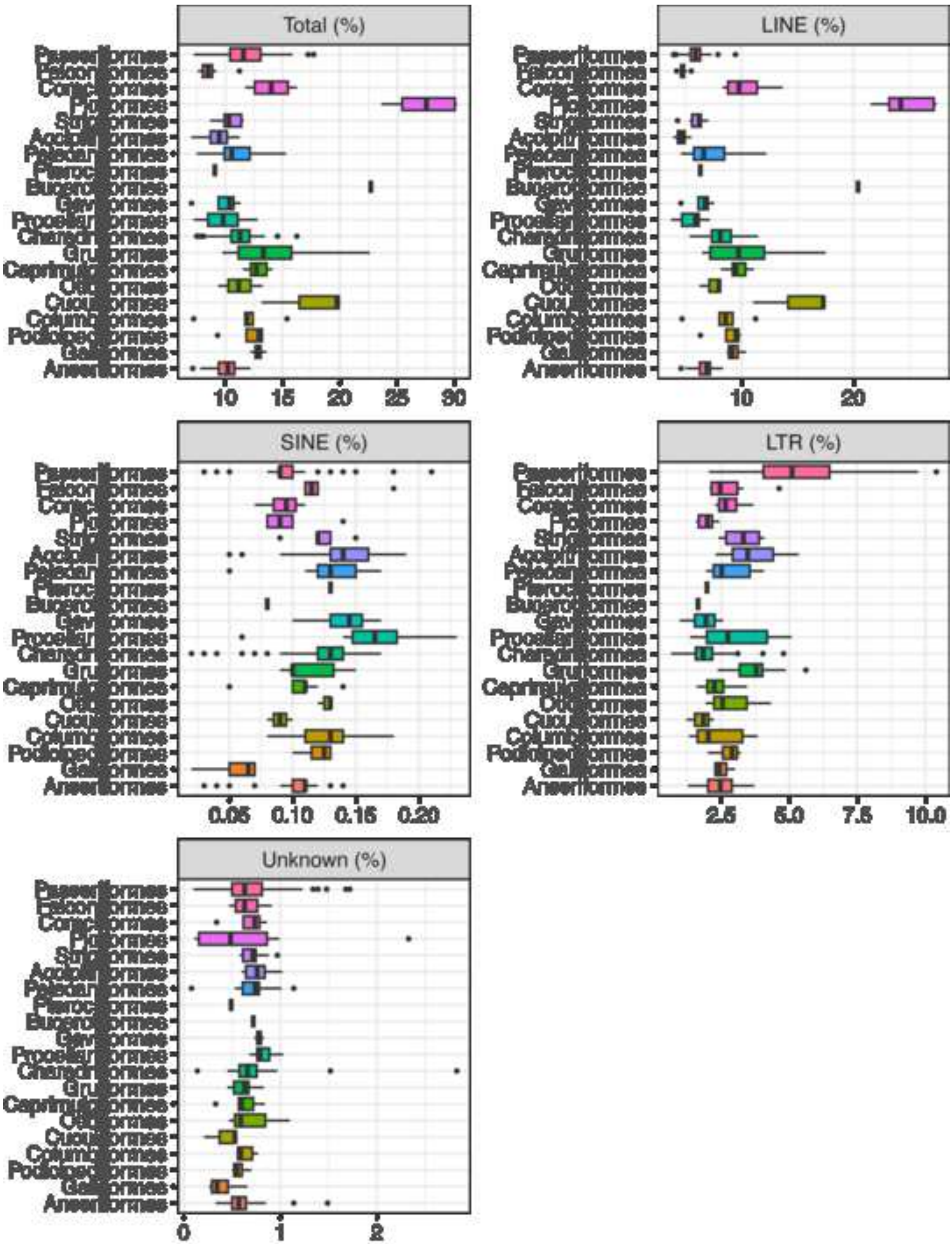

Table S1:Sample metadata

Click here to access/download
Supplementary Material
DanishBirdGenomeMetadata_sub_vers_20260519.xlsx

GIGA-D-25-00401

Dear Dr. Dongi Ma

Please see the newly revised version of our manuscript "A comprehensive reference genome assembly dataset of birds inhabiting Denmark, Greenland, and the Faroe Islands" (GIGA-D-25-00401). We appreciate the constructive feedback provided by reviewers, which has improved the manuscript and data description. In this letter, we describe the changes made to address reviewer and editor comments, which are in red. In summary, we have clarified the number of newly released vs. re-processed genomes, updating metadata with NCBI accession numbers and sex determination details, and added contamination screening information and quality control metrics (QV/K-mer).

Reviewer reports:
Reviewer #1: This paper serves primarily as an announcement of the data release for many new draft genome assemblies for most of the bird species that occur in Denmark, Greenland and/or the Faroe Islands. The paper includes the relevant sequencing and genome assembly methods along with various data summaries that provide some sense of the range of assembly quality and completeness. As the authors suggest, the data will be valuable for comparative genomic analyses and as a resource that facilitates population genetic/genomic studies of individual species. Public release of the data is clearly a good thing, and the manuscript appears to provide all information necessary to support this.

While there is little scope for asking the authors to change or augment the work already completed, I would be interested in hearing more from the authors on their recommendations for future work.

We included a summary of the recommended future use of the genomes, the first paragraph of the "Resuse potential" section.

Suppl. Fig. 3 highlights the fact that a subset of other currently available genomes are of higher quality, as measured by contig and scaffold N50 values. With advances in long-read technology and methods such as Hi-C, it has become relatively easy to generate higher quality assemblies with chromosome-length scaffolds. This raises a question about how best to allocate limited resources in future genome sequencing efforts… should the B10K effort continue to churn out draft genome assemblies for most birds as quickly as possible, or should efforts shift towards investing a bit more per species in the near term to achieve higher quality genomes that may have more lasting utility?

We discussed this topic briefly in paragraph 2 of "Re-use potential". The B10K project is archiving extracts and tissues to improve assemblies in the future, when the costs of adding these additional sequencing technologies come down.

I was pleased to note that the supplementary metadata spreadsheet included information about the sex of the samples used for sequencing. I was looking for information on this point in the main manuscript and think it would be helpful to let the reader know how many male and female samples were used with a note about the male samples yielding "incomplete" genomes without W chromosome data. Also, a minor point on that metadata spreadsheet: why was is not possible to determine sex using molecular data for all samples?

We have added a summary of the numbers of males and females sequenced, and added molecular sex determination for the missing species to the metadata file.

The text of the main paper was generally clear and easy to understand but I spotted a missing word or two in several sentences.

We have made several minor grammatical changes.

Reviewer #2: The authors present a substantial data set of 350 new short-read bird genome assemblies spanning the majority of avian diversity found in Denmark, Greenland, and the Faroe Islands. Although lagging current state of art in terms of the data types used and assembly quality (the assemblies fall short of current EBP recommendations), the sheer scale of the resource will be of great benefit to the research community and I am happy to recommend publication. Below some minor points to clarify and suggestions for small changes.

Abstract says they release 447 genomes but on reading the manuscript there are actually 350 newly released genomes and they have additionally processed 97 genomes that were previously released. I would update the abstract to better reflect the novel contribution of this data set which is substantial.

We have modified the abstract to clarify the number of new genomes released

The bioproject provided in the data availability section currently only has 151 assemblies associated with it. Are the rest of the genomes released elsewhere or just embargoed until publication?

These were awaiting acceptance at NCBI, and have been for almost a year. After this long delay, we re-uploaded the dataset to the ENA instead, and have added the new accession number to the manuscript.

Also, I couldn't see associated annotations with the assemblies. Possibly just not looking in the right place - are these available?

B10K Annotations are available on the B10K website, and the NCBI annotations can be found on ENA

It would also be great if the repeatmodeller libraries could be made public for each species as these are computationally intensive to produce.

We have added these to the B10K website.

It would be useful to state what proportion of the 350 genomes are for species with no previous genome assembly available. Ie. how novel is the data set?

All 350, at the time of submission.

To improve quality control, it would be good to see QV scores and Kmer-based completeness scores eg. with Merqury.

We have added these metrics to the metadata table.

There is no mention in the methods of how contamination was dealt with. Were contamination screens run?

We employed standard NCBI decontamination screens.

Re. Figure 3. The annotation statistics are not very informative on their own. It would be useful to compare the results for the new assemblies to a reference set of high-quality genome assemblies eg. take all long-read chromosome-scale bird genome assemblies, run the same pipeline and compare to the 350 new assemblies here. Or compare the annotation stats to NCBI refseq bird annotations. It would also be useful to compare BUSCO completeness of the gene sets to the BUSCO scores from the corresponding assembly to assess how well the annotation pipeline is performing.

We have added a statement comparing BUSCO scores to the avian genomes produced to VGP standards from Rhei et al.

Supp fig 3. The shading by assembly length is very difficult to interpret on the plot. If the purpose is to compare contiguity and length between new and published assemblies then a series of box plots / violin plots comparing the two sets for contig N50, scaffold N50 and assembly length would be clearer.

We have revised Figure 3 as described.

Please add assembly accession numbers to the supplementary table.

Done

Editor comments:

One reviewer has raised concerns regarding the authorship of the manuscript. Specifically, it appears that a number of listed authors may have been included primarily on the basis of having provided samples for sequencing. To address this concern, please ensure that the Author Contributions section clearly and transparently describes the specific contributions made by each listed author.

We have updated the contributions section. All authors who provided samples also collected and archived these resources, which is a highly skilled, expensive, and time-consuming endeavor. This is a 'substantial effort' as described in the authorship guidelines, as one of the most challenging parts of biodiversity genomics is sample acquisition.

We hope that this revision satisfies all issues raised by the reviewers and editors, and we look forward to your response.

On behalf of the B10K consortium members who contributed to this paper,
Sincerely,
Peter Hosner
