# Supplementary material for: A comprehensive reference genome assembly dataset of birds inhabiting Denmark, Greenland, and the Faroe Islands
Source: Gigascience. 2026 Jul 7;15:giag078. doi: 10.1093/gigascience/giag078 (PMC13492214; doi:10.1093/gigascience/giag078)
Supplement: Supplementary file 4 [file giag078_reviewer_1_report_original_submission.pdf]

Reviewer Report

Title: A comprehensive reference genome assembly dataset of birds inhabiting Denmark, Greenland, and the Faroe Islands

Version: Original Submission **Date:** 11/28/2025

Reviewer name: Reviewer 1

Reviewer Comments to Author:

This paper serves primarily as an announcement of the data release for many new draft genome assemblies for most of the bird species that occur in Denmark, Greenland and/or the Faroe Islands. The paper includes the relevant sequencing and genome assembly methods along with various data summaries that provide some sense of the range of assembly quality and completeness. As the authors suggest, the data will be valuable for comparative genomic analyses and as a resource that facilitates population genetic/genomic studies of individual species. Public release of the data is clearly a good thing, and the manuscript appears to provide all information necessary to support this.

While there is little scope for asking the authors to change or augment the work already completed, I would be interested in hearing more from the authors on their recommendations for future work. Suppl. Fig. 3 highlights the fact that a subset of other currently available genomes are of higher quality, as measured by contig and scaffold N50 values. With advances in long-read technology and methods such as Hi-C, it has become relatively easy to generate higher quality assemblies with chromosome-length scaffolds. This raises a question about how best to allocate limited resources in future genome sequencing efforts… should the B10K effort continue to churn out draft genome assemblies for most birds as quickly as possible, or should efforts shift towards investing a bit more per species in the near term to achieve higher quality genomes that may have more lasting utility?

I was pleased to note that the supplementary metadata spreadsheet included information about the sex of the samples used for sequencing. I was looking for information on this point in the main manuscript and think it would be helpful to let the reader know how many male and female samples were used with a note about the male samples yielding "incomplete" genomes without W chromosome data. Also, a minor point on that metadata spreadsheet: why was is not possible to determine sex using molecular data for all samples?

The text of the main paper was generally clear and easy to understand but I spotted a missing word or two in several sentences.

Methods

Are the methods appropriate to the aims of the study, are they well described, and are necessary controls included? Choose an item.

Conclusions

Are the conclusions adequately supported by the data shown? Choose an item.

Reporting Standards

Does the manuscript adhere to the journal's guidelines on minimum standards of reporting? Choose an item.

Choose an item.

Statistics

Are you able to assess all statistics in the manuscript, including the appropriateness of statistical tests used? Choose an item.

Quality of Written English

Please indicate the quality of language in the manuscript: Choose an item.

Declaration of Competing Interests

Please complete a declaration of competing interests, considering the following questions:

- Have you in the past five years received reimbursements, fees, funding, or salary from an organisation that may in any way gain or lose financially from the publication of this manuscript, either now or in the future?
- Do you hold any stocks or shares in an organisation that may in any way gain or lose financially from the publication of this manuscript, either now or in the future?
- Do you hold or are you currently applying for any patents relating to the content of the manuscript?
- Have you received reimbursements, fees, funding, or salary from an organization that holds or has applied for patents relating to the content of the manuscript?
- Do you have any other financial competing interests?
- Do you have any non-financial competing interests in relation to this paper?

If you can answer no to all of the above, write 'I declare that I have no competing interests' below. If your reply is yes to any, please give details below.

I declare that I have no competing interests

I agree to the open peer review policy of the journal. I understand that my name will be included on my report to the authors and, if the manuscript is accepted for publication, my named report including any attachments I upload will be posted on the website along with the authors' responses. I agree for my report to be made available under an Open Access Creative Commons CC-BY license (http://creativecommons.org/licenses/by/4.0/). I understand that any comments which I do not wish to be included in my named report can be included as confidential comments to the editors, which will not be published.

Choose an item.

To further support our reviewers, we have joined with Publons, where you can gain additional credit to further highlight your hard work (see: https://publons.com/journal/530/gigascience). On publication of this paper, your review will be automatically added to Publons, you can then choose whether or not to claim your Publons credit. I understand this statement.

Yes Choose an item.
