# Supplementary material for: A comprehensive reference genome assembly dataset of birds inhabiting Denmark, Greenland, and the Faroe Islands
Source: Gigascience. 2026 Jul 7;15:giag078. doi: 10.1093/gigascience/giag078 (PMC13492214; doi:10.1093/gigascience/giag078)
Supplement: Supplementary file 5 [file giag078_reviewer_2_report_original_submission.pdf]

Reviewer Report

Title: A comprehensive reference genome assembly dataset of birds inhabiting Denmark, Greenland, and the Faroe Islands

Version: Original Submission **Date:** 12/17/2025

Reviewer name: Reviewer 2

Reviewer Comments to Author:

The authors present a substantial data set of 350 new short-read bird genome assemblies spanning the majority of avian diversity found in Denmark, Greenland, and the Faroe Islands. Although lagging current state of art in terms of the data types used and assembly quality (the assemblies fall short of current EBP recommendations), the sheer scale of the resource will be of great benefit to the research community and I am happy to recommend publication. Below some minor points to clarify and suggestions for small changes.

Abstract says they release 447 genomes but on reading the manuscript there are actually 350 newly released genomes and they have additionally processed 97 genomes that were previously released. I would update the abstract to better reflect the novel contribution of this data set which is substantial.

The bioproject provided in the data availability section currently only has 151 assemblies associated with it. Are the rest of the genomes released elsewhere or just embargoed until publication? Also, I couldn't see associated annotations with the assemblies. Possibly just not looking in the right place - are these available? It would also be great if the repeatmodeller libraries could be made public for each species as these are computationally intensive to produce.

It would be useful to state what proportion of the 350 genomes are for species with no previous genome assembly available. Ie. how novel is the data set?

To improve quality control, it would be good to see QV scores and Kmer-based completeness scores eg. with Merqury.

There is no mention in the methods of how contamination was dealt with. Were contamination screens run?

Re. Figure 3. The annotation statistics are not very informative on their own. It would be useful to compare the results for the new assemblies to a reference set of high-quality genome assemblies eg. take all long-read chromosome-scale bird genome assemblies, run the same pipeline and compare to the 350 new assemblies here. Or compare the annotation stats to NCBI refseq bird annotations. It would also be useful to compare BUSCO completeness of the gene sets to the BUSCO scores from the corresponding assembly to assess how well the annotation pipeline is performing.

Supp fig 3. The shading by assembly length is very difficult to interpret on the plot. If the purpose is to compare contiguity and length between new and published assemblies then a series of box plots / violin plots comparing the two sets for contig N50, scaffold N50 and assembly length would be clearer.

Please add assembly accession numbers to the supplementary table.

Methods

Are the methods appropriate to the aims of the study, are they well described, and are necessary controls included? Choose an item.

Conclusions

Are the conclusions adequately supported by the data shown? Choose an item.

Reporting Standards

Does the manuscript adhere to the journal's guidelines on minimum standards of reporting? Choose an item.

Choose an item.

Statistics

Are you able to assess all statistics in the manuscript, including the appropriateness of statistical tests used? Choose an item.

Quality of Written English

Please indicate the quality of language in the manuscript: Choose an item.

Declaration of Competing Interests

Please complete a declaration of competing interests, considering the following questions:

- Have you in the past five years received reimbursements, fees, funding, or salary from an organisation that may in any way gain or lose financially from the publication of this manuscript, either now or in the future?
- Do you hold any stocks or shares in an organisation that may in any way gain or lose financially from the publication of this manuscript, either now or in the future?
- Do you hold or are you currently applying for any patents relating to the content of the manuscript?
- Have you received reimbursements, fees, funding, or salary from an organization that holds or has applied for patents relating to the content of the manuscript?
- Do you have any other financial competing interests?
- Do you have any non-financial competing interests in relation to this paper?

If you can answer no to all of the above, write 'I declare that I have no competing interests' below. If your reply is yes to any, please give details below.

I declare that I have no competing interests.

I agree to the open peer review policy of the journal. I understand that my name will be included on my report to the authors and, if the manuscript is accepted for publication, my named report including any attachments I upload will be posted on the website along with the authors' responses. I agree for my report to be made available under an Open Access Creative Commons CC-BY license (http://creativecommons.org/licenses/by/4.0/). I understand that any comments which I do not wish to be included in my named report can be included as confidential comments to the editors, which will not be published.

Choose an item.

To further support our reviewers, we have joined with Publons, where you can gain additional credit to further highlight your hard work (see: https://publons.com/journal/530/gigascience). On publication of this paper, your review will be automatically added to Publons, you can then choose whether or not to claim your Publons credit. I understand this statement.

Yes Choose an item.
