# Supplementary material for: A comprehensive reference genome assembly dataset of birds inhabiting Denmark, Greenland, and the Faroe Islands
Source: Gigascience. 2026 Jul 7;15:giag078. doi: 10.1093/gigascience/giag078 (PMC13492214; doi:10.1093/gigascience/giag078)
Supplement: Supplementary file 6 [file giag078_supplemental_files.zip › Supplementary Figure 1.pdf]

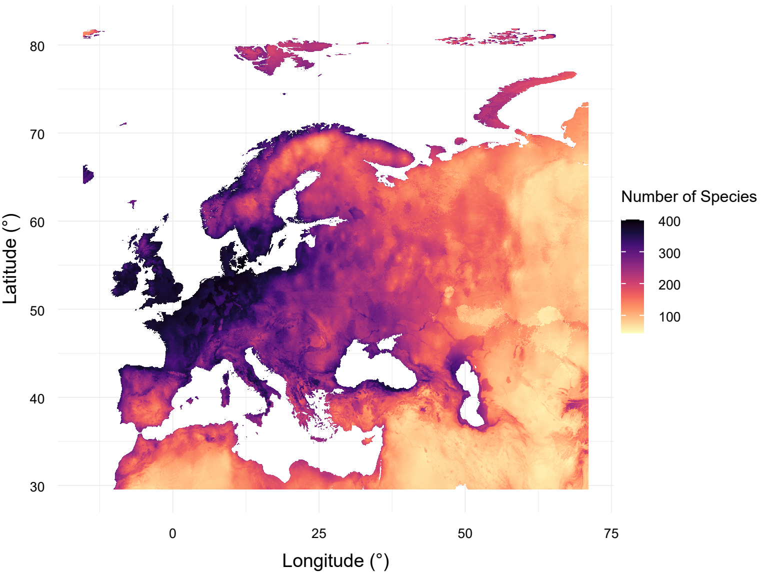
80
70
60
50
40
30
Latitude (°)
0
25
50
75
Longitude (°)
Number of Species
400
300
200
100
